# The brain creates illusions not just for us: sharks (*Chiloscyllium griseum*) can “see the magic” as well

**DOI:** 10.3389/fncir.2014.00024

**Published:** 2014-03-20

**Authors:** Theodora Fuss, Horst Bleckmann, Vera Schluessel

**Affiliations:** Department for Comparative Sensory Biology and Neurobiology, Institute of Zoology, Rheinische Friedrich-Wilhelms-University BonnBonn, Germany

**Keywords:** optical illusion, Kanizsa, subjective contour, Müller-Lyer deception, elasmobranch, *Chiloscyllium griseum*

## Abstract

Bamboo sharks (*Chiloscyllium griseum*) were tested for their ability to perceive subjective and illusionary contours as well as line length illusions. Individuals were first trained to differentiate between squares, triangles, and rhomboids in a series of two alternative forced-choice experiments. Transfer tests then elucidated whether Kanizsa squares and triangles, grating gaps and phase shifted abutting gratings were also perceived and distinguished. The visual systems of most vertebrates and even invertebrates perceive illusionary contours despite the absence of physical luminance, color or textural differences. Sharks are no exception to the rule; all tasks were successfully mastered within 3–24 training sessions, with sharks discriminating between various sets of Kanizsa figures and alternative stimuli, as well as between subjective contours in >75% of all tests. However, in contrast to Kanizsa figures and subjective contours, sharks were not deceived by Müller-Lyer (ML) illusions. Here, two center lines of equal length are comparatively set between two arrowheads or –tails, in which case the line featuring the two arrow tails appears to be longer to most humans, primates and birds. In preparation for this experiment, lines of varying length, and lines of unequal length randomly featuring either two arrowheads or -tails on their ends, were presented first. Both sets of lines were successfully distinguished by most sharks. However, during presentation of the ML illusions sharks failed to succeed and succumbed either to side preferences or chose according to chance.

## Introduction

**Figure 1:**
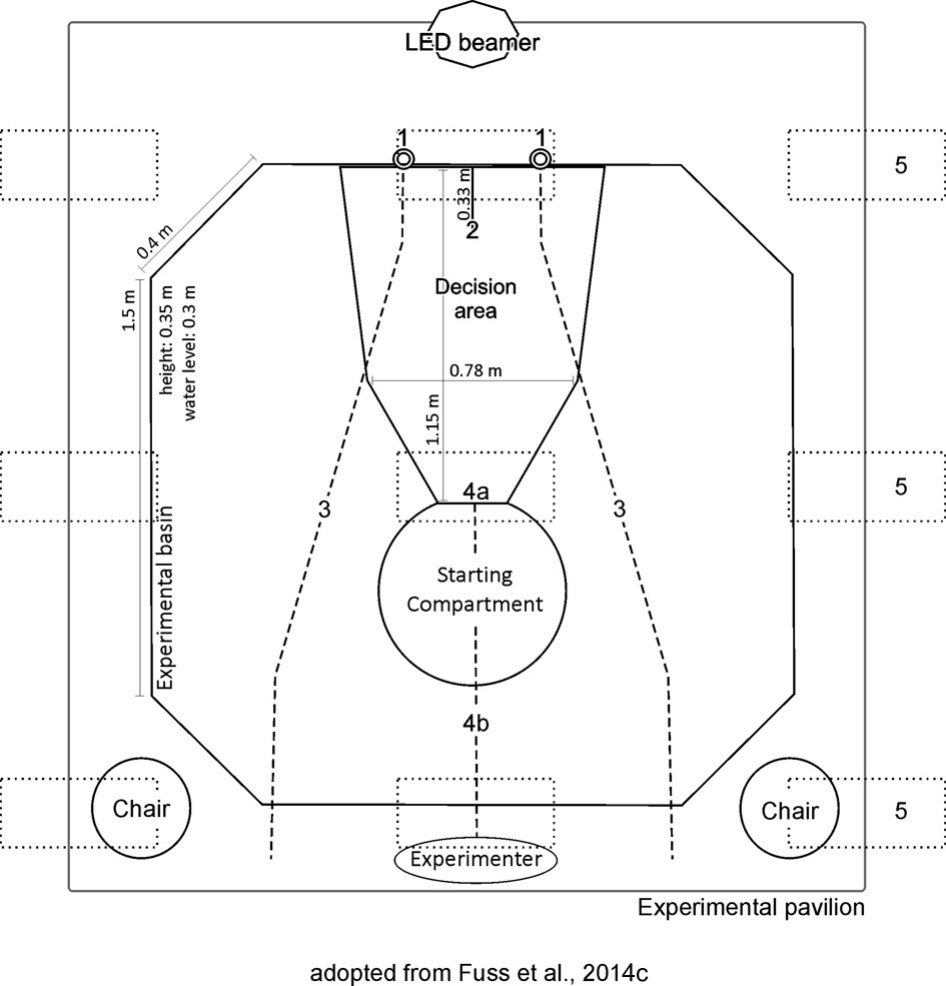
**The experimental setup located within the experimental basin, inside the white pavilion.** The keyhole-shaped setup consisted of a Starting Compartment, a decision area and a frosted screen for projections, featuring a divider allowing for unambiguous choice-making (left and right). For the projections, a LED beamer was used. Sharks were placed within the SC at the start of each trial. 1 = feeders, 2 = frosted screen for projection, 3 = cable pulls to release feeders, 4a = guillotine door, 4b = cable pull to open guillotine door, 5 = ceiling mounted fluorescent tubes (above pavilion roof).

Illusionary contours, such as Kanizsa squares or triangles are misreadings of visual information by the brain; instead of processing merely the actual information coming from the retina, the brain adheres to preconceptions and assumes what is most likely to be seen, based on previous experiences and neural wiring (Kandel et al., 2000). In this respect, vision is a creative, interactive process that depends on both the real properties of a visual object as well as contextual interactions and prior experiences, which are organized by processing different pieces of information (e.g., shape or color) according to system specific rules (Kandel et al., 2000). The most famous examples for such phenomena are provided by the “Kanizsa figures,” which are produced when the brain is fooled into seeing a square or a triangle, without there actually being a physical counterpart (Kanizsa, 1974). The triangle-illusion for example, is created by the arrangement of three Pacmen figures positioned with their open angles of 60° all pointing inwards to the same region (see Figure 1). In the absence of any lines or color changes, this arrangement itself is sufficient to evoke the impression in the viewer of there being distinct contours forming a triangle. This impression is strengthened by the fact that the illusionary triangle also appears to be brighter than the background despite a homogenous luminance. In the field of Gestalt psychology, Kanizsa figures and other illusions are explained using the principle that the brain first assesses objects as a whole or an entity prior to or instead of paying attention to individual components or parts. Additionally, if parts are lacking and an object is incomplete an entirety will be imagined whenever possible. Accordingly, objects that are close together also tend to be perceived as belonging together.

Several studies have shown that teleosts, like mammals, birds and even insects, can be deceived by optical illusions (e.g., Nieder, 2002; Agrillo et al., 2013), perceive illusionary contours, e.g., Kanizsa figures (Wyzisk, 2005; Wyzisk and Neumeyer, 2007) and can recognize partly occluded or fragmented objects (Sovrano and Bisazza, 2008, 2009; Darmaillacq et al., 2011). Very recently, a review on illusionary contours in teleosts was published by Agrillo et al. (2013) but so far, the ability to perceive illusionary contours has not been tested in any elasmobranch (sharks and rays). Elasmobranchs belong to the class Chondrichthyes (cartilaginous fishes), which represents the oldest extant jawed vertebrates. Recent research has finally been shedding light onto the previously neglected and often disputed cognitive abilities within this group, specifically in regards to learning and memory. Results indicate that the once popular disclaimer “primitive fish with primitive brains” is well and truly out of date and that sharks and rays can solve many cognitive tasks to the same extent as other vertebrates (Reviewed by Guttridge et al., 2010; Schluessel and Bleckmann, 2005, 2012; Kuba et al., 2010; Spaet et al., 2010; Schwarze et al., 2013; Fuss et al., 2014a,b,c; Kimber et al., 2014). Nonetheless, many questions regarding cognition in elasmobranchs still remain unanswered, whereas cognition in teleosts has been studied in much more detail and has been summarized in a detailed review by Brown et al. (2011). This study aimed to determine if the shark brain can be deceived by optical illusions, i.e., if it follows the same rules and principles in regards to the creative vision process as other vertebrate brains. The ability to perceive illusionary contours that lack a physical counterpart (Petry and Meyer, 1987; Schumann, 1900) shows that the visual system contains inferences about the world beyond available sensory information—whether on low levels (Paradiso et al., 1989; von der Heydt, 1995) or on a cognitive basis (Gregory, 1972; Rock and Anson, 1979). Accordingly, optical illusions can provide valuable information on the processing of sensory stimuli in the brain and the neural basis of form vision.

Three experiments were conducted to test the perception of illusionary contours in sharks, i.e., (1) Kanizsa figures, (2) Subjective contours, and (3) Müller-Lyer (ML) illusions. Gray bamboo sharks (*Chiloscyllium griseum*) are small, benthic sharks that naturally occur in the Indo-West Pacific (Compagno et al., 2005). They primarily inhabit shallow waters, such as lagoons and inshore environments, sea grass meadows as well as rocky and coral reef environments, occupy small territories and feed on benthic prey (Compagno et al., 2005). Sharks are more distantly related to teleosts and other vertebrates than birds are to mammals or mammals are to each other and shark brains are very differently organized and structured from teleost brains due to divergent developmental processing (Northcutt, 1977; Wullimann and Mueller, 2004; Nieuwenhuys, 2009). Experiments were therefore aimed to allow for new insights into the processing of (subjective) sensory information in the brain in one of the most ancient vertebrate groups.

## Materials and methods

### Animals and housing facilities

Nine juvenile bamboo sharks (*Chiloscyllium griseum*, 4 male, 5 female, TL: 25–40 cm) were kept in aquaria (1 × 0.5 × 0.5 m) connected to each other and to the experimental setup, providing constant environmental conditions (conductivity, temperature, and pH). The system was filled with aerated, filtered salt water [conductance: about 50 mS (ca. 1,0217 kg/dm^3^)] at 26 ± 2°C. Food (small pieces of squid, fish, or shrimp) was only available during the experimental training. Experiments were conducted during daylight hours; there was a 12 h light: 12 h dark cycle. Individuals were identified by phenotypic characteristics.

### Set-up

Experiments were performed by using the same octagonal experimental basin as well as the same setup as outlined previously (Fuss et al., 2014c). The gray PVC setup (Figure 1) featured a Starting Compartment (SC, 0.51 × 0.35 m), a decision area (113.5 × 0.87 × 0.35 m) and a frosted screen for projection (0.92 × 0.35 m) and was placed within an octagonal experimental basin (2.5 × 2.5 × 0.35 m) made out of transparent Perspex featuring a white covered floor (Figure 1). During experiments, the basin was filled with water to a depth of about 0.3 m. To exclude uncontrolled cueing as well as other potentially disturbing external influences, the basin was surrounded by a white pavilion (3.0 × 3.0 × 2.5 m). Ceiling mounted fluorescent tubes allowed an even illumination during the experiments (above pavilion roof; Osram L 18 W, Lumilux Cool White, Germany).

A light gray guillotine door (0.43 × 0.23 m) confined the SC (0.43 × 0.3 × 0.35 m), in which sharks were placed before each trial. Independent of the type of trial/experiment the experimenter was situated behind the SC. The guillotine door was controlled manually by using a cable pull. A 0.33 m long divider, attached to the frosted screen separated a left from a right division, thereby allowing for an unambiguous decision making in response to the two stimuli displayed on the screen (Figure 1) via a projector. For projections, a LED projector situated at a distance of 1.3 m from the screen was used (Figure 1). The bluish-green colored stimuli used during all experiments were displayed on a light gray colored background. According to Hart et al. (2011), the maximum absorbance (λ_max_) of cone visual pigments in the very closely related shark species *Chiloscyllium punctatum* was found at 531.8 ± 6.7 nm; in the visible light range for blue to green. As sharks were usually swimming close to the bottom, stimuli were projected at a height of 3 cm above the ground. To reward sharks for a correct decision, feeders were installed just above both stimuli, which allowed food to be dropped into the setup manually using a cable pull from the experimenter's position at the opposite side of the experimental set up (Figure 1). For a correct choice to be recorded by the experimenter, sharks had to press their nose against the wall just below/onto the positive stimulus. Selected sessions were videotaped. Both feeders were baited during all trials to exclude unintentional cueing. Additionally, the water in the maze was stirred after every trial to preclude any olfactory cues after a reward was given (which could bias the shark's choice of arm in subsequent trials).

### Training

Training followed the schedule outlined previously (Fuss et al., 2014c). The behavioral experiments consisted of three phases: 1—acclimatization, 2—training (regular trials), and 3—transfer trials. Experiments were conducted as two-alternative-forced-choice experiments. After successful training of the first stimulus set (phase 1), performance was tested in the remaining pairs.

#### Phase 1—acclimatization

Before training, sharks were allowed to become familiar with the experimental setup by swimming freely throughout the entire setup for up to 20 min at a time. The guillotine door was open, both divisions displayed the same 2D object (circle) and feeders were in place. Once a shark swam freely throughout the maze and looked for food being dropped from the feeders (i.e., nearby the 2D objects), training commenced (Figure 1).

#### Phase 2—training

**Figure 2:**
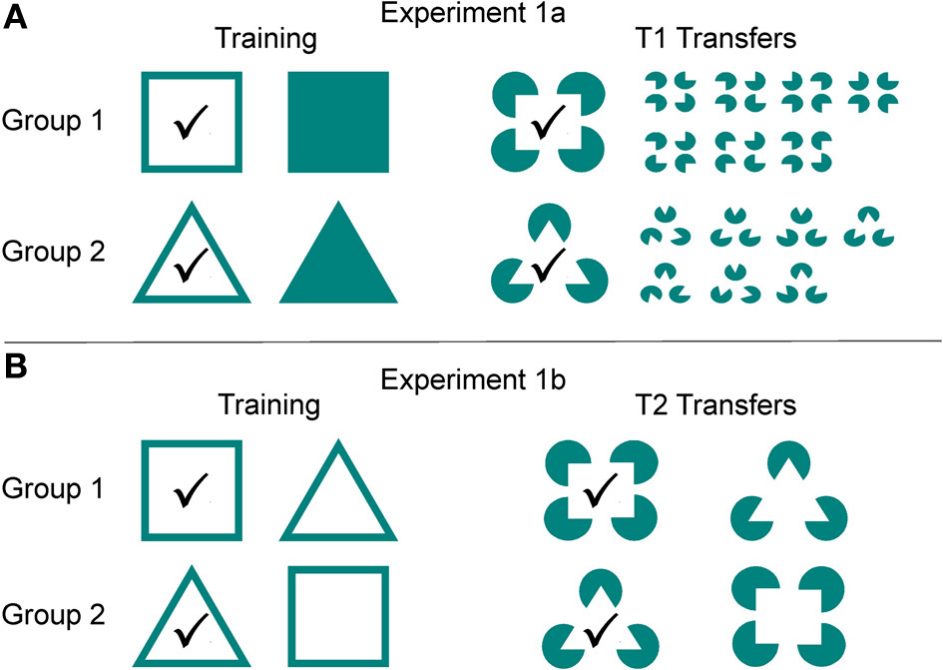
**Experiment 1.** Shown are the stimuli that were presented to each group during regular training and transfer test trials in experiments 1a and 1b. The positive, rewarded stimulus is indicated by a checkmark. **(A)** In group 1 an empty square was the positive, rewarded stimulus, in group 2 it was an empty triangle. During the T1 transfer tests of experiment 1a, sharks were “expected” to choose the correct Kanizsa figure. **(B)** During experiment 1b, group 1 was trained to recognize an empty square over an empty triangle, whereas group 2 was trained vice versa. During the T2 transfer tests, sharks were expected to choose the Kanizsa figure resembling the stimulus they had been trained on.

**Figure 3:**
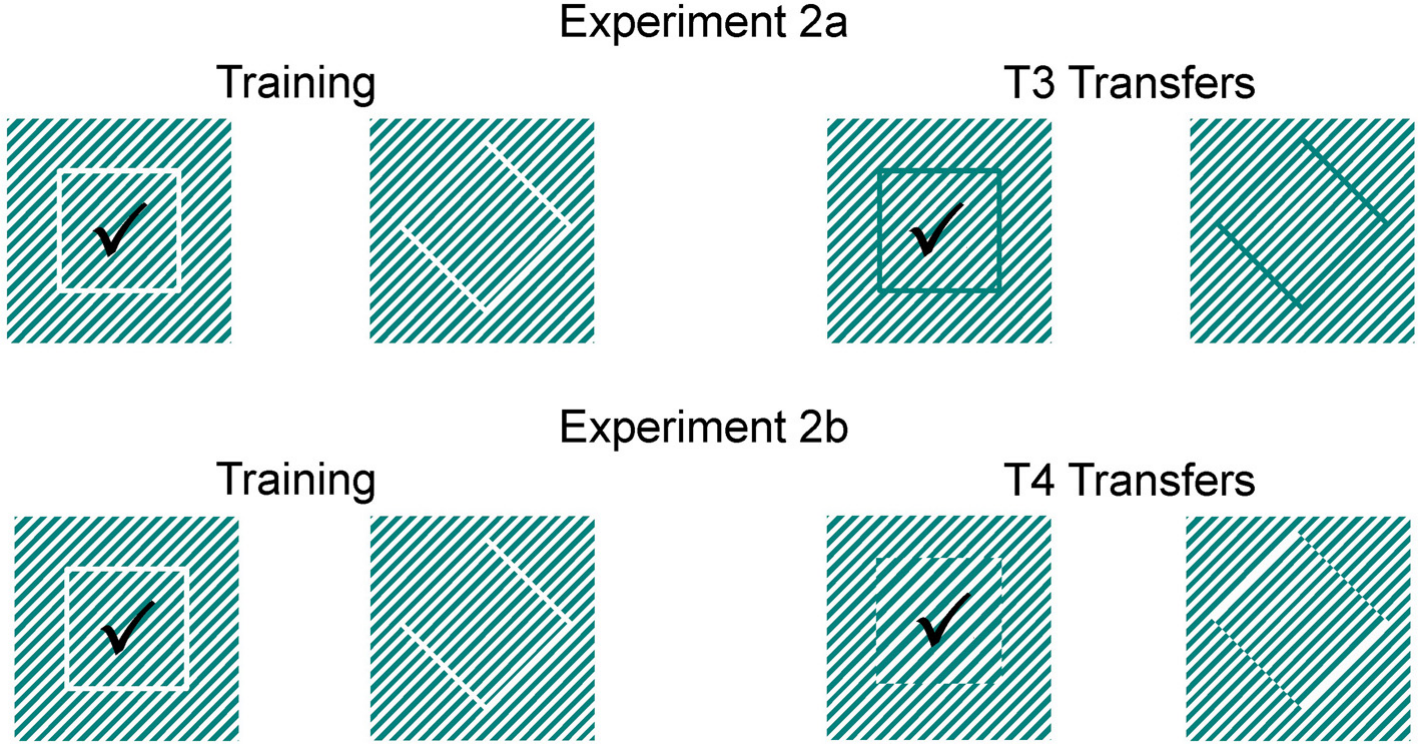
**Experiment 2.** Shown are the stimuli presented to each group during regular training and transfer test trials. The positive, rewarded stimulus is marked by a checkmark. All sharks were trained to choose a white square presented on diagonal lines. During T3 transfer tests (2a), sharks were expected to choose the subjective contour defining a square by using grating gaps within the white lines; during T4 transfer tests (2b), sharks were expected to choose the subjective contour defining a square by using phase-shifted abutting gratings.

**Figure 4:**
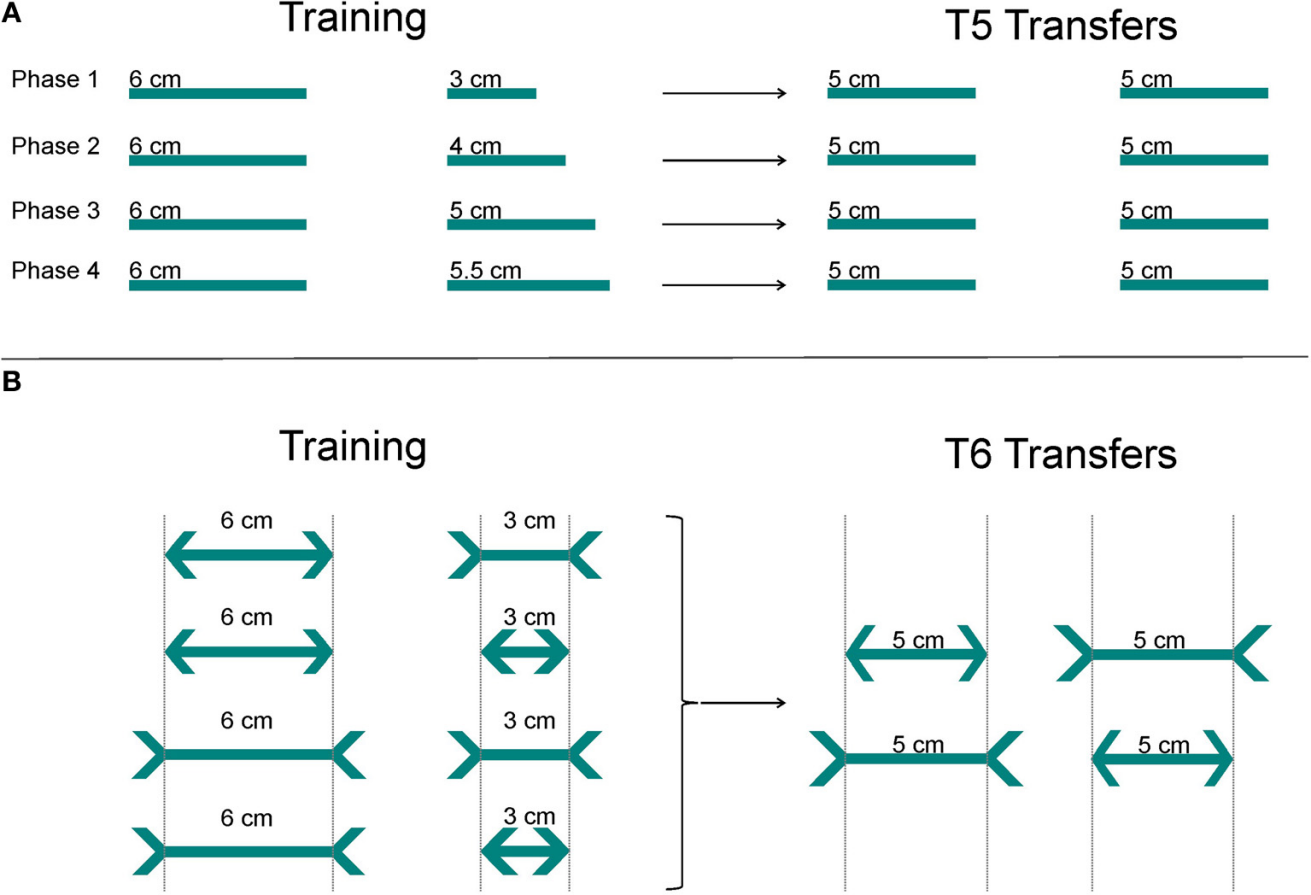
**Experiment 3.** Shown are the stimuli presented to each group during the regular training and transfer test trials (3a and 3b). The positive, rewarded stimulus is marked by a checkmark. **(A)** In 3a, all sharks were trained to choose the longer of the two lines. In the T5 transfer tests, sharks were presented with two lines of equal length (5 vs. 5 cm). **(B)** In the second part (3b), sharks were trained to choose the longer of the two lines, irrespective of the orientation of the arrowheads (arrowheads or -tails). During the T6 transfer tests, sharks were presented with the Müller-Lyer deception (two lines of equal length but with differently oriented arrowheads). The gray dotted lines are only shown here to simplify the figure, but were not shown during the experiments.

Before each trial, both feeders were baited and the water stirred. At the beginning of each regular trial the shark was placed in the SC. To start a trial, the shark had to push against the guillotine door with its snout. A trial lasted for a maximum of 2 min. A choice was made as soon as the shark touched the frosted screen on the opposite end of the set up with its snout. The two stimuli (Figures 2–4) to be discriminated were displayed simultaneously (one in each division) and switched randomly between the left and the right side of the screen (Figure 1) to avoid direction conditioning. Five alternating rotational schemes were used, so as to vary the succession of stimuli shown on a particular side between sessions. A correct choice was rewarded with food. During the inter-trial-interval (ITI), the shark was allowed to swim freely throughout the entire setup for 30 s, before it was gently guided back into the SC. The next trial started as soon as the shark pushed against the guillotine door. If a shark did not choose within the allocated 2 min, the trial was terminated. Training sessions were carried out 5 days per week; each session consisted of ten trials. Training was completed as soon as a learning criterion of ≥70% correct choices on three subsequent sessions was reached (χ^2^(1) ≤ 0.05; to prove statistical significance). If an animal did not reach the criterion within 30 training sessions it was excluded from further training.

#### Phase 3—transfers

Transfer tests were conducted during which the sharks had to perform under altered conditions. Up to two transfer trials were interspersed randomly with ten regular trials within one session and separated by at least five regular trials from each other (resulting in 12 trials per session). Transfer trials remained unrewarded to prevent any kind of learning with respect to the new situation. During this phase, a maximum of eight regular trials (out of ten) were rewarded (random selection) irrespective of choice. This served to prepare the fish for transfer trials (so as to keep the fish from realizing that only transfer trials were unrewarded and therefore not worth participating in).

### Experiment 1: Kanizsa figures

#### Experiment 1a

During training, there were two groups: group 1 (*n* = 4) learned to recognize an empty square as the positive, rewarded stimulus over a filled one, whereas group 2 (*n* = 4) learned to recognize an empty triangle over a filled one (Figure 2). As soon as the learning criterion was reached, the transfer phase commenced. During the transfer tests (T1) it was tested if sharks preferentially chose the Kanizsa figure (group 1: resembling a square, group 2: resembling a triangle) over seven different randomized Pacmen figures (Figure 2). Each shark participated in 28 transfer tests.

#### Experiment 1b

Group 1 (*n* = 3) was trained to recognize an empty square as the positive, rewarded stimulus over an empty triangle, whereas Group 2 (*n* = 4) was trained to recognize an empty triangle over an empty square. Following successful training, sharks were presented with a series of 28 transfer tests (T2) with the aim to determine whether the Kanizsa figure resembling the positive stimulus during regular trials (group 1: a square, group 2: a triangle) was chosen over the alternative one (Figure 2).

### Experiment 2: subjective contours

All sharks (*n* = 8) were trained to choose a white square presented on diagonal lines (Figure 3), while the negative stimulus was a rhomboid. Following successful training, sharks were presented with subjective contours in a series of transfer tests. During 30 transfer tests of experiment 2a (T3) a correct choice was recorded, if the subjective contour defining a square by using grating gaps within the white lines was chosen over a rhomboid (Figure 3). In experiment 2b, sharks were then presented with a second series of 30 transfer tests (T4) and tested if the subjective contour defining a square by using phase-shifted abutting gratings was chosen over the subjective contour defining a rhomboid (Figure 3).

### Experiment 3: size ratios and Müller-Lyer deception

#### Experiment 3a

All sharks (*n* = 8) learned to distinguish between two lines of different lengths (6 vs. 3 cm, 6 vs. 4 cm, 6 vs. 5 cm, 6 vs. 5.5 cm; see Figure 4). The longer of the two lines served as the positive, rewarded stimulus. Following successful training on the first pair, sharks were presented with a series of ten transfer tests (T5) before they continued with training of the next pair. Transfer tests of experiment 3a (5 vs. 5 cm) served to test whether other cues aside from the length of the lines helped the shark to recognize the positive stimulus and to determine behavior (Figure 4).

#### Experiment 3b

All sharks (*n* = 8) learned to distinguish between two unequally sized lines (center line 6 vs. 3 cm) equipped with either arrow “heads” or arrow “tails” (i.e., “correct” or “inverted” arrowheads, see Figure 4). The longer line served as the positive, rewarded stimulus. The line length and orientation of arrowheads switched randomly between the left and the right side of the screen (Figure 4). Transfer tests (T6) were performed to test whether sharks are deceived by ML illusions. Accordingly, in these transfer test trials sharks were presented with two center lines of equal length (5 cm) but with differently oriented arrowheads (Figure 4). Each shark participated in 30 transfer tests.

### Data analysis

The average trial time, the percentage of correct choices and the percentage of right and left choices were recorded for each session for each individual. A Chi^2^ test was performed to test for significant side preferences of individuals. To prove statistical significance of learning success, the learning criterion was established to be ≥70% correct choices in three consecutive sessions (χ^2^(1) ≤ 0.05). A sign and binomial test was run to determine if those sharks, who did not reach the learning criterion within 30 sessions still chose the positive (rewarded) stimulus significantly more often than the negative (unrewarded) stimulus. A Mann-Whitney-U test was used to determine if the average trial times differed significantly between the regular training trials and the transfer test trials for each individual as well as for groups. Sign and binomial tests as well as the 95% confidence intervals of a proportion (both by using the absolute numbers of decisions) were calculated for each individual as well as for the group(s) to determine whether sharks preferred one symbol or one side significantly over the other. To test for differences between the two groups (experiment 1), a Wilcoxon signed rank test was used. For all tests a *p* ≤ 0.05 was considered significant, a *p* ≤ 0.001 highly significant.

## Results

Nine sharks participated in the experimental training procedure (Shark 1 died after experiment 1a and was replaced by Shark 9 at the beginning of experiment 2). The following section will summarize individual results for those nine sharks as well as for the group. Group results include only those sharks, which finished a phase successfully.

### Acclimatization

Sharks (*n* = 9) needed on average 11.22 ± 3.27 sessions to acclimatize to the maze, perform the starting procedure and retrieve food from the feeders. Initial side preferences were only observed in one individual [χ^2^_Shark1_(1) = 0.014, χ^2^_Shark2_(1) = 0.295, χ^2^_Shark3_(1) = 0.604, χ^2^_Shark4_(1) = 0.795, χ^2^_Shark5_(1) = 0.188, χ^2^_Shark6_(1) = 0.434, χ^2^_Shark7_(1) = 0.796, χ^2^_Shark8_(1) = 1, χ^2^_Shark9_(1) = 0.604].

### Experiment 1: Kanizsa figures

**Figure 5:**
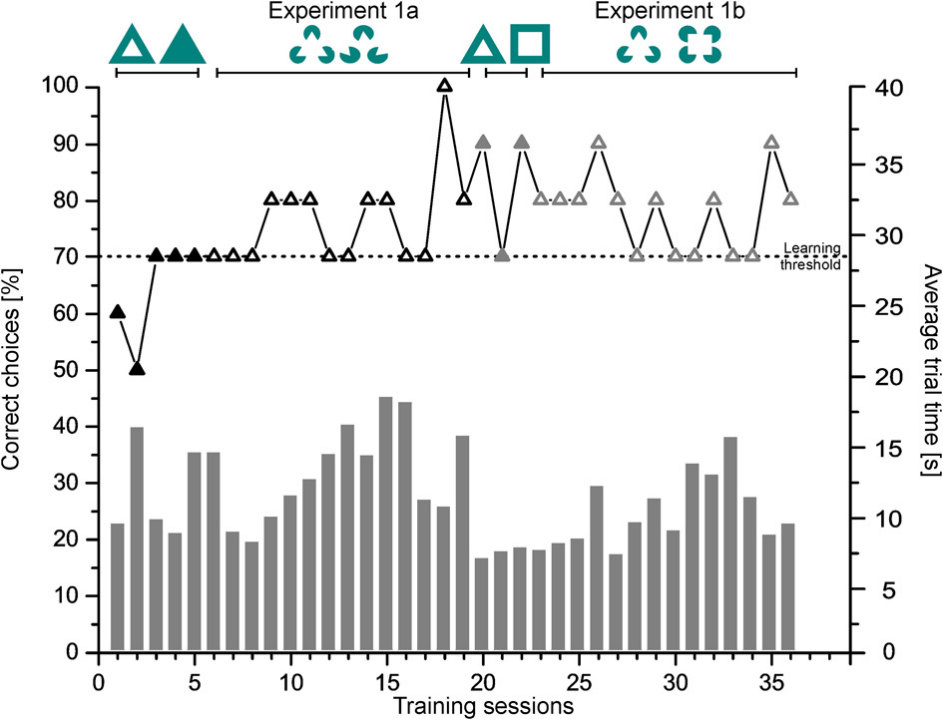
**Experiment 1.** Shown is the performance of Shark 7 as % of correct choices (symbolized by triangles; left ordinate) per session as well as the average trial time in seconds (symbolized by gray bars; right ordinate) per session per phase until the learning criterion was reached.

**Figure 6:**
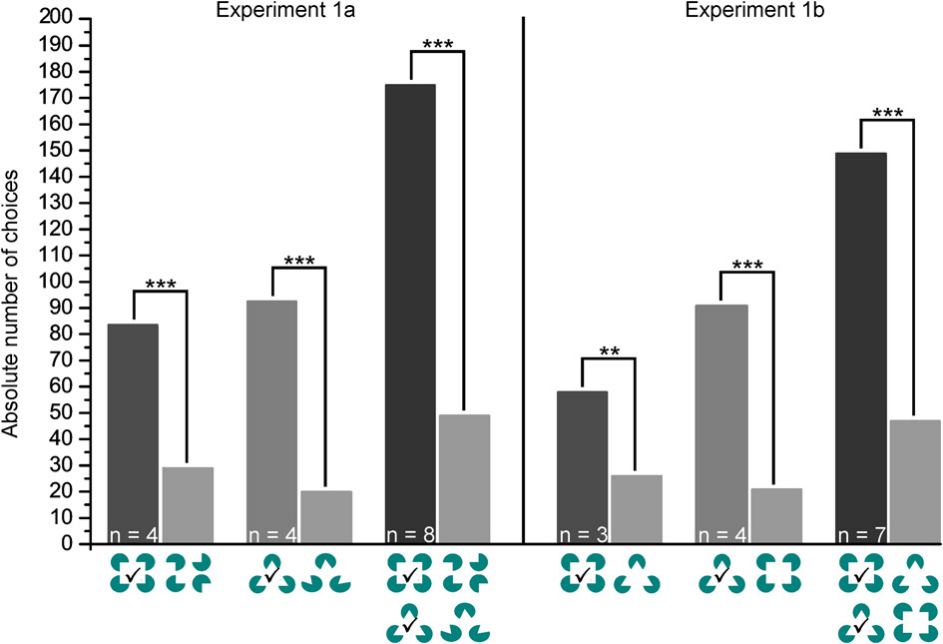
**Experiment 1a and b.** Shown are the group results for the transfer test trials. *p* > 0.05 not significant, *p* ≤ 0.01 significant (^**^), *p* ≤ 0.001 significant (^***^). The correct choice is marked by a checkmark.

In Figure 5 a representative learning curve of one individual (Shark 7) is provided for the different phases of experiment 1 until the learning criterion was reached. Additionally, average trial time per session is given in seconds. Group results of the transfer trials during experiment 1a and 1b are summarized in Figure 6.

#### Experiment 1a

Sharks needed on average 10.13 ± 6.29 sessions (group 1: 8.00 ± 2.45, group 2: 12.25 ± 8.62) to complete training successfully. On average 10.90 ± 2.24 s per trial were needed (group 1: 12.15 ± 4.15s, group 2: 10.17 ± 2.58 s) to make a decision (for individual details please compare Table 1).

Table 1**Part 1: Statistics on the *performance* during regular training trials and transfer tests during Experiment 1: Kanizsa figures (Experiments 1a and 1b)**.

**Table:** 

**Subject**	**Experiment 1a**				**Experiment 1b**			
	**Sessions to reach learning criterion**	**Correct vs. incorrect choices during transfers (T1)**	**Sign and binomial test on correct choices during transfers (T1)**	**The 95% confidence interval extends from**	**Sessions to reach learning criterion**	**Correct vs. incorrect choices during transfers (T2)**	**Sign and binomial test on correct choices during transfers (T2)**	**The 95% confidence interval extends from**
Group 1	8 ± 2.45	83: 29	One-tail: *p* ≤ 0.001^***^	0.652–0.814	7.33 ± 6.66	58: 26	One-tail: *p* ≤ 0.001^***^	0.545–0.779
			Two-tail: *p* ≤ 0.001^***^				Two-tail: *p* ≤ 0.001^***^	
Shark 1^#^	8	21: 7	One-tail: *p* = 0.006^**^	0.564–0.876	–	–	–	–
			Two-tail: *p* = 0.012^*^					
Shark 2	11	22: 6	One-tail: *p* = 0.002^**^	0.601–0.901	3	21: 7	One-tail: *p* = 0.006^**^	0.564–0.876
			Two-tail: *p* = 0.004^**^				Two-tail: *p* = 0.012^*^	
Shark 3	5	17: 11	One-tail: *p* = 0.173	0.424–0.765	4	17: 11	One-tail: *p* = 0.172	0.424–0.765
			Two-tail: *p* = 0.345				Two-tail: *p* = 0.345	
Shark 4	8	23: 5	One-tail: *p* ≤ 0.001^***^	0.639–0.926	15	20: 8	One-tail: *p* ≤ 0.018^**^	0.527–0.849
			Two-tail: *p* ≤ 0.001^***^				Two-tail: *p* ≤ 0.036^*^	
Group 2	12.25 ± 8.62	92: 20	One-tail: *p* ≤ 0.001^***^	0.739–0.882	7.75 ± 5.25	91: 21	One-tail: *p* ≤ 0.001^***^	0.729–0.874
			Two-tail: *p* ≤ 0.001^***^				Two-tail: *p* ≤ 0.001^***^	
Shark 5	22	23: 5	One-tail: *p* ≤ 0.001^***^	0.639–0.926	8	23: 5	One-tail: *p* ≤ 0.001^***^	0.639–0.926
			Two-tail: *p* ≤ 0.001^***^				Two-tail: *p* ≤ 0.001^***^	
Shark 6	5	21: 7	One-tail: *p* = 0.006^**^	0.564–0.876	5	22: 6	One-tail: *p* = 0.002^**^	0.601–0.901
			Two-tail: *p* = 0.012^**^				Two-tail: *p* = 0.004^**^	
Shark 7	5	24: 4	One-tail: *p* ≤ 0.001^***^	0.679–0.949	3	22: 6	One-tail: *p* ≤ 0.001^***^	0.601–0.901
			Two-tail: *p* ≤ 0.001^***^				Two-tail: *p* ≤ 0.001^***^	
Shark 8	17	24: 4	One-tail: *p* ≤ 0.001^***^	0.679–0.949	15	24: 4	One-tail: *p* = 0.002^**^	0.679–0.949
			Two-tail: *p* ≤ 0.001^***^				Two-tail: *p* = 0.004^**^	
Group 1 + 2	10.13 ± 6.29	175: 49	One-tail: *p* ≤ 0.001^***^	0.722–0.831	7.57 ± 5.35	149: 47	One-tail: *p* ≤ 0.001^***^	0.696–0.815
			Two-tail: *p* ≤ 0.001^***^				Two-tail: *p* ≤ 0.001^***^	

p > 0.05 not significant,

(*) p ≤ 0.05 significant

(**) p ≤ 0.01 significant

(***) *p ≤ 0.001 significant*.

# Shark 1 died between Experiment 1a and 1b and did therefore not participate in Experiment 1b.

**Table:** **Part 2: Statistics on the *average trial times* [s] during regular training trials and transfer tests during Experiment 1: Kanizsa figures (Experiments 1a and 1b)**.

**Subject**	**Experiment 1a**				**Experiment 1b**			
	**Average trial time [s] per regular training trial during training (Tr) and transfer (Tr1)**		**Average trial time [s] per transfer trial (T1)**	**Mann-Whitney-U test on time differences [training (Tr1) ↔ transfers (T1)]**	**Average trial time [s] per regular training trial during training (Tr) and transfer (Tr2)**		**Average trial time [s] per transfer trial (T2)**	**Mann-Whitney-U test on time differences [training (Tr2) ↔ transfers (T2)]**
	**Tr**	**Tr1**			**Tr**	**Tr2**		
Group 1	12.15 ± 4.15 s	11.16 ± 4.68 s	12.81 ± 10.94 s	*Z* = 1.571 *p* = 0.116	11.80 ± 4.92 s	9.74 ± 3.27 s	11.36 ± 9.68 s	*Z* = 0.887 *p* = 0.375
Shark 1^#^	10.49 ± 2.39 s	12.35 ± 4.67 s	19.50 ± 16.61 s	*Z* = −0.199 *p* = 0.842	−	−	−	–
Shark 2	8.40 ± 1.6 s	6.69 ± 1.0 s	7.50 ± 3.7 s	*Z* = −0.294 *p* = 0.768	5.60 ± 0.36 s	7.01 ± 1.39 s	7.32 ± 4.51 s	*Z* = 0.602 *p* = 0.847
Shark 3	13.14 ± 3.15 s	10.48 ± 2.46 s	10.43 ± 4.39 s	*Z* = 0.575 *p* = 0.566	7.89 ± 1.18 s	9.36 ± 1.82 s	9.36 ± 3.16 s	*Z* = 0.754 *p* = 0.451
Shark 4	14.90 ± 4.08 s	14.63 ± 5.16 s	13.82 ± 10.02 s	*Z* = 1.709 *p* = 0.087	14.06 ± 4.26 s	12.87 ± 3.22 s	17.39 ± 14.10 s	*Z* = −0.160 *p* = 0.873
Group 2	10.17 ± 2.58 s	11.27 ± 4.37 s	11.54 ± 10.34 s	*Z* = 2.837 *p* = 0.005^**^	8.90 ± 2.39	9.44 ± 3.27 s	9.86 ± 7.54 s	*Z* = 1.989 *p* = 0.047^*^
Shark 5	10.01 ± 2.91 s	11.46 ± 4.55 s	10.36 ± 7.04 s	*Z* = 1.635 *p* = 0.102	8.10 ± 2.28 s	8.79 ± 1.95 s	8.86 ± 5.90 s	*Z* = 1.814 *p* = 0.069
Shark 6	8.36 ± 1.54 s	7.50 ± 1.29 s	7.29 ± 4.53 s	*Z* = 1.702 *p* = 0.089	8.92 ± 1.58 s	9.30 ± 3.34 s	8.82 ± 8.01 s	*Z* = 1.472 *p* = 0.141
Shark 7	11.42 ± 3.44 s	12.88 ± 3.34 s	16.29 ± 16.10 s	*Z* = 1.136 *p* = 0.256	7.03 ± 0.40 s	10.00 ± 2.55 s	10.54 ± 9.05 s	*Z* = 1.017 *p* = 0.309
Shark 8	10.52 ± 2.01 s	13.17 ± 4.79 s	12.21 ± 8.11 s	*Z* = 1.329 *p* = 0.184	10.01 ± 2.51 s	9.65 ± 2.70 s	11.30 ± 6.89 s	*Z* = −0.079 *p* = 0.937
Group 1 + 2	10.9 ± 3.59 s	11.27 ± 4.51 s	12.17 ± 10.64 s	*Z* = 3.082 *p* = 0.002^**^	9.95 ± 3.83 s	9.57 ± 2.92 s	10.51 ± 8.54 s	*Z* = 2.134 *p* = 0.033^*^

p > 0.05 not significant,

(*) p ≤ 0.05 significant

(**) p ≤ 0.01 significant

# Shark 1 died between Experiment 1a and 1b and did therefore not participate in Experiment 1b.

During transfer tests, all but one shark (Shark 3, Table 1) chose the “correct” figure (the corresponding Kanizsa figure) significantly more often than the incorrect one (Table 1). All sharks solved the T1 transfer tests on average within 12.17 ± 10.64 s per trial (Table 1). This was not significantly different from the regular training trials during the transfer test phase, neither for any individual nor for group 1 (Table 1). In group 2, there were no significant differences between the regular and transfer trials for any individual but for the group as a whole (Table 1).

#### Experiment 1b

Sharks needed on average 7.57 ± 5.35 sessions (group 1: 7.33 ± 6.66, group 2: 7.75 ± 5.25) to complete training successfully (for individual details please compare Table 1). On average 9.95 ± 3.83 s per trial were needed (group 1: 11.80 ± 4.92 s, group 2: 8.90 ± 2.39 s) to make a decision.

The whole group solved the T2 transfer tests on average within 10.51 ± 8.54 s per transfer trial (Table 1). During transfer tests, all but one shark (Shark 3, Table 1) chose the correct figure significantly more often than the incorrect one (Table 1). Sharks needed on average 10.51 ± 8.54 s per transfer trial (Table 1). This was not significantly different from the regular training trials during the transfer test phase, neither for any individual nor for group 1 (Table 1). In group 2, no significant differences were found in the performance of individual sharks but for the group as a whole (Table 1).

In comparison, there was no significant difference between group 1 and group 2 in the absolute number of correct choices during transfer test trials between Experiment 1a and 1b (NPH two samples: *Z* = −1.323, *p* = 0.186; Wilcoxon signed rank test: *Z* = −1.105, *p* = 0.375). Additionally, there was no significant difference in the average trial time to solve the regular training trials or the transfer test trials for any shark, but for group 2 as well as for all sharks combined (Table 1).

### Experiment 2: subjective contours

**Figure 7:**
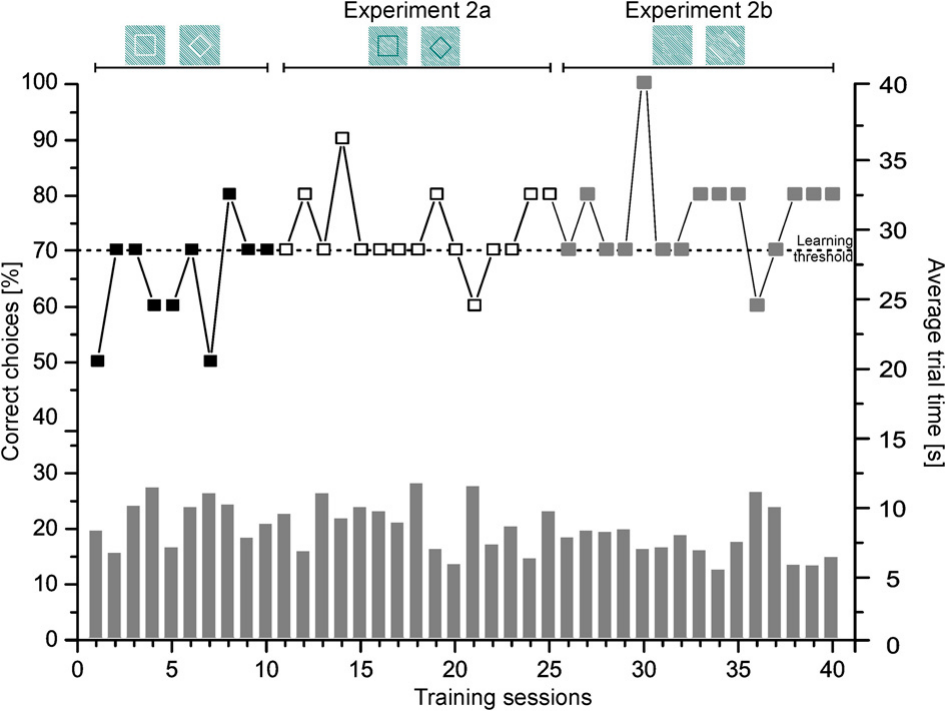
**Experiment 2.** Shown is the performance of Shark 8 as % of correct choices per session (symbolized by boxes; left ordinate) as well as the average trial time (s) per session (symbolized by gray bars; right ordinate) per phase until the learning criterion was reached.

**Figure 8:**
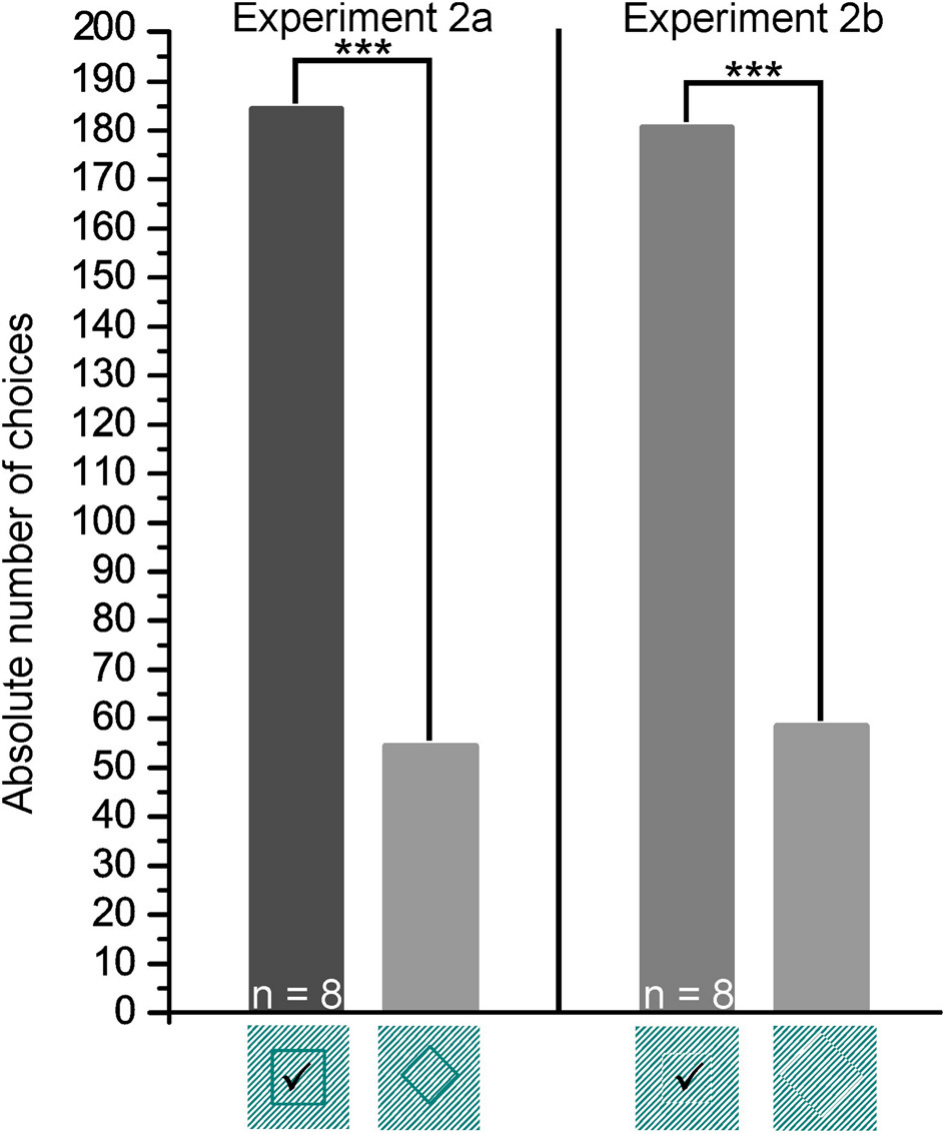
**Experiment 2.** Shown are the group results for the transfer trials (2a and 2b). *p* > 0.05 not significant, *p* ≤ 0.001 significant (^***^). The correct choice is marked by a checkmark.

Figure 7 provides a representative learning curve of one individual (Shark 8) for the different phases of experiment 2 until the learning criterion was reached. Additionally, the average trial time per session is given in seconds. Group results of the transfer trials during Experiment 2a and 2b are summarized in Figure 8.

Sharks needed on average 11.13 ± 8.44 sessions to complete training successfully (Table 2). They needed on average 9.88 ± 3.48 s per training trial to make a decision.

Table 2**Part 1: Statistics on the *performance* during regular training trials and transfer tests during Experiment 2: subjective contours**.

**Table:** 

**Subject**	**Sessions to reach learning criterion**	**Correct vs. incorrect choices during transfers (T3)**	**Sign and binomial test on correct choices during transfers (T3)**	**The 95% confidence interval extends from**	**Correct vs. incorrect choices during transfers (T4)**	**The 95% confidence interval extends from**	**Sign and binomial test on correct choices during transfers (T4)**
Shark 2	24	21: 9	One-tail *p* = 0.021^*^	0.519–0.835	23: 7	0.588–0.885	One-tail *p* = 0.003^**^
			Two-tail *p* = 0.043^*^				Two-tail *p* = 0.005^**^
Shark 3	24	22: 8	One-tail *p* = 0.008^**^	0.553–0.860	22: 8	0.554–0.860	One-tail *p* = 0.008^**^
			Two-tail *p* = 0.016^*^				Two-tail *p* = 0.016^*^
Shark 4	4	23: 7	One-tail *p* = 0.002^**^	0.588–0.885	23: 7	0.588–0.885	One-tail *p* = 0.003^**^
			Two-tail *p* = 0.005^**^				Two-tail *p* = 0.005^**^
Shark 5	3	24: 6	One-tail *p* ≤ 0.001^***^	0.623–0.909	22: 8	0.554–0.860	One-tail *p* = 0.008^**^
			Two-tail *p* = 0.001^***^				Two-tail *p* = 0.016^*^
Shark 6	7	24: 6	One-tail *p* ≤ 0.001^***^	0.623–0.909	24: 6	0.623–0.909	One-tail *p* ≤ 0.001^***^
			Two-tail *p* = 0.001^***^				Two-tail *p* = 0.001^***^
Shark 7	3	26: 4	One-tail *p* ≤ 0.001^***^	0.697–0.953	26: 4	0.697–0.953	One-tail *p* ≤ 0.001^***^
			Two-tail *p* ≤ 0.001^***^				Two-tail *p* ≤ 0.001^***^
Shark 8	10	25: 5	One-tail *p* ≤ 0.001^***^	0.659–0.931	21: 9	0.519–0.835	One-tail *p* = 0.021^*^
			Two-tail *p* ≤ 0.001^***^				Two-tail *p* = 0.043^*^
Shark 9	17	20: 10	One-tail *p* = 0.049^*^	0.487–0.809	20: 10	0.467–0.809	One-tail *p* = 0.049^*^
			Two-tail *p* = 0.099				Two-tail *p* = 0.098
Group	11.13 ± 8.44	185: 55	One-tail *p* ≤ 0.001^***^	0.713–0.819	181: 59	0.696–0.804	One-tail *p* ≤ 0.001^***^
			Two-tail *p* ≤ 0.001^***^				Two-tail *p* ≤ 0.001^***^

p > 0.05 not significant,

(*) p ≤ 0.05 significant

(**) p ≤ 0.01 significant

(***) *p ≤ 0.001 significant*.

**Table:** **Part 2: Statistics on the *average trial time* [s] during regular training trials and transfer tests during Experiment 2: subjective contours**.

**Subject**	**Average trial time [s] per regular training trial during training (Tr), transfer T3, transfer T4**			**Average trial time [s] per transfer trial**		**Mann-Whitney-U test on time differences [training (TrT3) ↔ transfers (T3) and training (TrT4) ↔ transfers (T4)]**	
	**Tr**	**T3**	**T4**	**T3**	**T4**	**TrT3 T3**	**TrT4 T4**
Shark 2	7.75 ± 1.98 s	7.21 ± 1.78 s	7.33 ± 1.55 s	8.67 ± 5.96 s	8.03 ± 3.87 s	*Z* = 0.544 *p* = 0.586	*Z* = 0.396 *p* = 0.692
Shark 3	10.15 ± 2.11 s	10.54 ± 2.12 s	11.64 ± 2.21 s	11.60 ± 6.61 s	14.13 ± 5.93 s	*Z* = 0.479 *p* = 0.631	*Z* = −0.681 *p* = 0.495
Shark 4	12.50 ± 2.79 s	15.84 ± 4.68 s	14.06 ± 4.34 s	20.03 ± 26.47 s	14.13 ± 12.79 s	*Z* = 1.420 *p* = 0.155	*Z* = 1.222 *p* = 0.222
Shark 5	9.30 ± 1.59 s	11.39 ± 4.47 s	10.27 ± 3.24 s	14.13 ± 14.21 s	11.63 ± 9.07 s	*Z* = 0.443 *p* = 0.658	*Z* = 1.207 *p* = 0.227
Shark 6	6.59 ± 1.16 s	7.46 ± 2.47 s	8.21 ± 2.61 s	10.30 ± 16.78 s	7.93 ± 3.72 s	*Z* = 1.012 *p* = 0.311	*Z* = 0.671 *p* = 0.502
Shark 7	8.80 ± 2.66 s	8.36 ± 1.39 s	9.05 ± 2.62 s	10.70 ± 5.36 s	10.73 ± 6.65 s	*Z* = −1.038 *p* = 0.299	*Z* = 0.084 *p* = 0.933
Shark 8	8.54 ± 1.64 s	8.27 ± 1.86 s	6.99 ± 1.53 s	8.00 ± 6.58 s	6.1 s ± 3.42 s	*Z* = 1.692 *p* = 0.091	*Z* = 1.806 *p* = 0.071
Shark 9	14.39 ± 3.78 s	19.35 ± 6.63 s	16.50 ± 5.60 s	18.53 ± 8.47 s	16.63 ± 13.43 s	*Z* = 0.543 *p* = 0.587	*Z* = 1.398 *p* = 0.162
Group	9.88 ± 3.48 s	10.02 ± 4.16 s	9.96 ± 3.67 s	12.57 ± 14.94	11.10 ± 7.99 s	*Z* = 0.666 *p* = 0.505	*Z* = 0.774 *p* = 0.438

p > 0.05 not significant.

All sharks solved the T3 transfer tests on average within 12.57 ± 14.94 s per trial, T4 transfer tests on average within 11.10 ± 7.99 s per trial. During transfer tests, all but one shark (Shark 9, Table 2) chose the correct figure (the corresponding square) significantly more often than the incorrect one (Table 2). There was no significant difference regarding the average trial time of regular vs. transfer test trials (T3 and T4; Table 2) for any shark.

### Experiment 3: size ratios and Müller-Lyer deception

**Figure 9:**
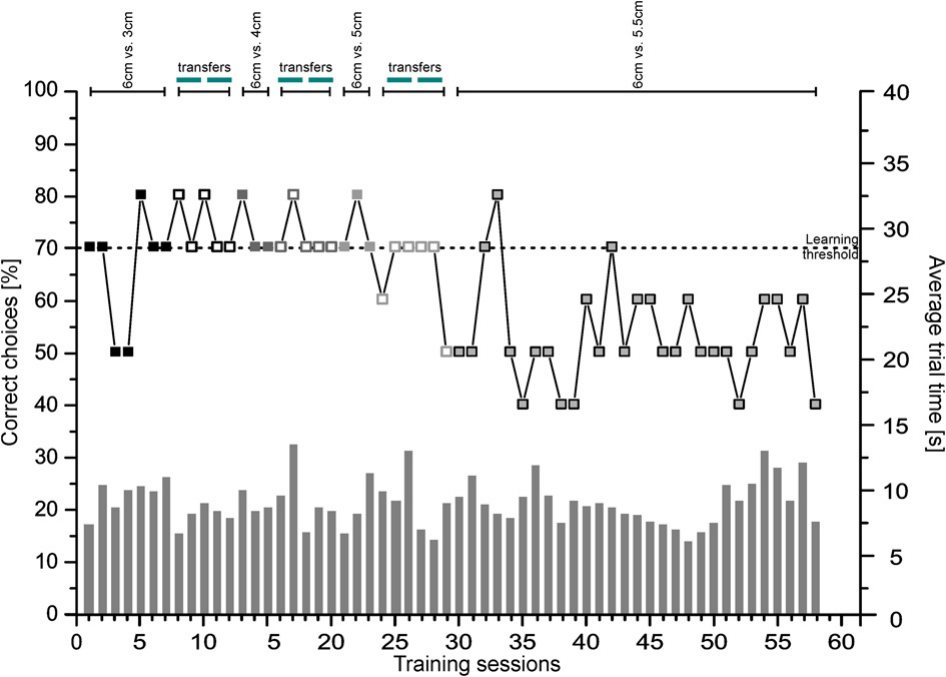
**Size pairs.** Shown is the performance of Shark 3 as the percentage of correct choices per session (symbolized by boxes; left ordinate) as well as the average trial time (s) per session (symbolized by gray bars; right ordinate) per phase until the learning criterion was reached.

**Figure 10:**
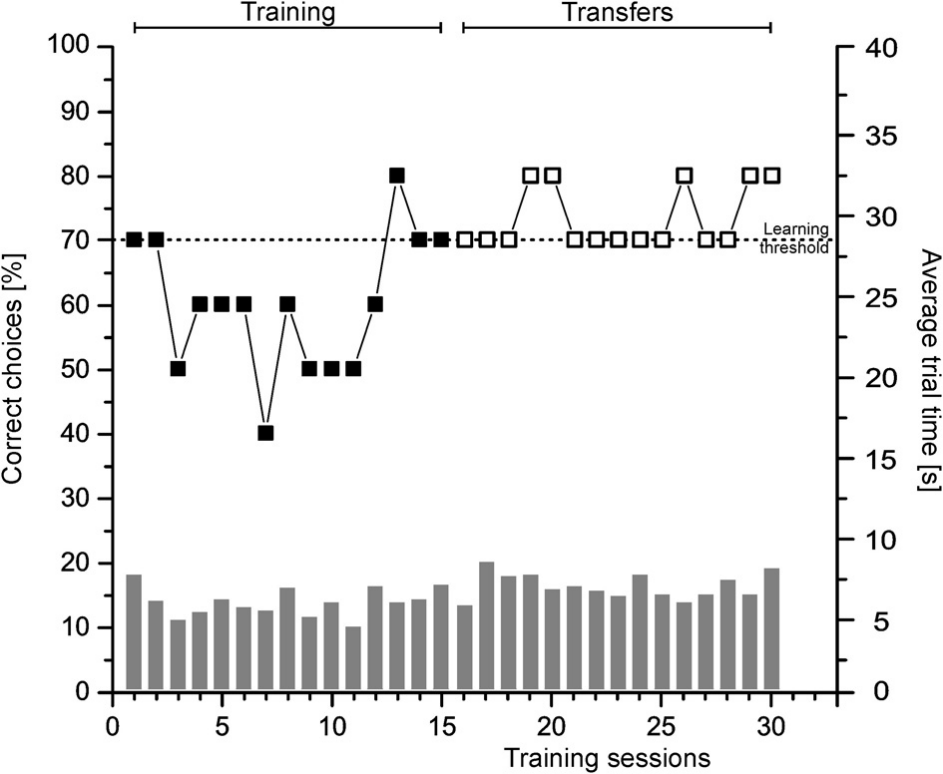
**Müller-Lyer deception.** Shown is the performance of Shark 5 as the percentage of correct choices per session (symbolized by boxes; left ordinate) as well as the average trial time (s) per session (symbolized by gray bars; right ordinate) per phase until the learning criterion was reached.

**Figure 11:**
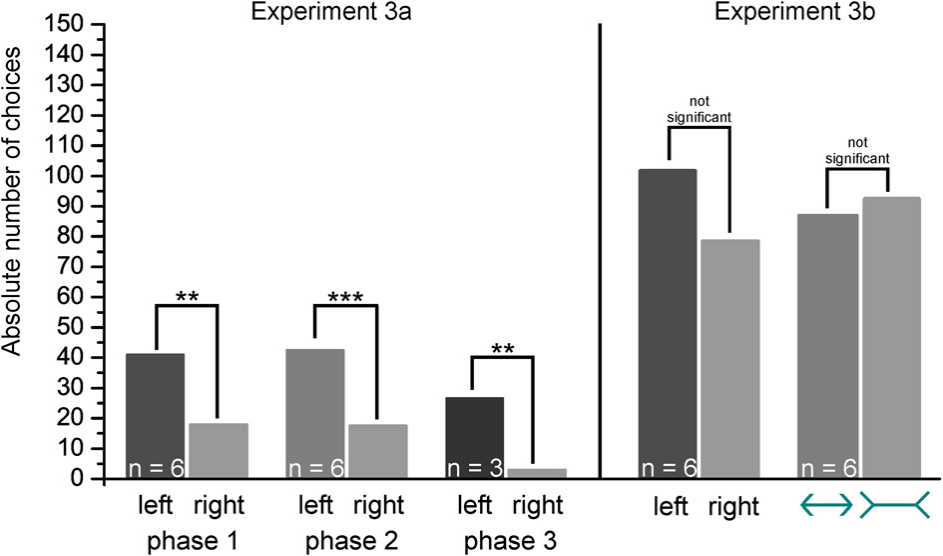
**Size pairs and Müller-Lyer deception.** Shown are the group results of the transfer test trials. *p* > 0.05 not significant, *p* ≤ 0.01 highly significant (^**^), *p* ≤ 0.001 significant (^***^). The correct choice is marked by a checkmark.

In Figures 9, 10 representative learning curves of two individuals (Figure 9: Shark 3, Figure 10: Shark 5) are provided for the different phases of experiment 3 until the learning criterion was reached. Additionally, the average trial time per session is given in seconds. Group results of the transfer trials during Experiment 3a and 3b are summarized in Figure 11 (for individual details please compare Tables 3, 4).

Table 3**Part 1: Statistics on regular training trails and transfer tests during Experiment 3: Size pairs and Müller-Lyer deception (Experiments 3a and 3b)**.

**Table:** 

**Subject^#^**	**Experiment 3a**							
	**Sessions to reach learning criterion**	**Average trial time [s] per regular training trial during training (Tr) and transfer (Tr5)**		**“Left” vs. “right” choices during transfers (T5)**	**Sign and binomial test on side preferences during transfers (T5)**	**The 95% confidence interval extends from**	**Average trial time [s] per transfer trial (T5)**	**Mann-Whitney-U test on time differences (training (Tr5) ↔ transfers (T5)**
		**Tr**	**Tr5**					
**6 vs. 3 cm**								
Shark 2	9	6.69 ± 1.30 s	6.74 ± 1.727 s	7: 3	One-tail *p* = 0.172	0.392–0.897	5.80 ± 1.549 s	*Z* = 1.116 *p* = 0.265
					Two-tail *p* = 0.344			
Shark 3	7	9.17 ± 9.17 s	7.54 ± 6.00 s	6: 4	One-tail *p* = 0.377	0.312–0.833	6.00 ± 1.49 s	*Z* = 1.923 *p* = 0.055
					Two-tail *p* = 0.754			
Shark 5	4	6.96 ± 0.98 s	9.82 ± 6.90 s	8: 2	One-tail *p* = 0.055	0.479–0.954	6.90 ± 4.82 s	*Z* = 1.671 *p* = 0.095
					Two-tail *p* = 0.109			
Shark 6	3	7.47 ± 1.45 s	6.74 ± 1.24 s	10: 0	One-tail *p* = 0.001^***^	0.679–1.000	4.80 ± 1.55 s	*Z* = 2.301 *p* = 0.022^*^
					Two-tail *p* = 0.002^**^			
Shark 7	3	7.83 ± 0.32 s	11.98 ± 5.96 s	6: 4	One-tail *p* = 0.377	0.312–0.833	7.80 ± 3.19 s	*Z* = 1.671 *p* = 0.095
					Two-tail *p* = 0.754			
Shark 8	11	6.34 ± 0.71 s	6.02 ± 0.72 s	4: 6	One-tail *p* = 0.377	0.167–0.688	6.20 ± 2.25 s	*Z* = −0.616 *p* = 0.538
					Two-tail *p* = 0.754			
Group	6.17 ± 3.37	7.20 ± 1.44 s	8.14 ± 3.85 s	41: 19	One-tail *p* = 0.003^**^	0.557–0.787	6.25 ± 2.79 s	*Z* = 4.001 *p* ≤ 0.001^***^
					Two-tail *p* = 0.006^**^			
**6 vs. 4 cm**								
Shark 2	8	6.20 ± 0.99 s	7.43 ± 1.42 s	7: 3	One-tail *p* = 0.172	0.392–0.897	5.30 ± 0.95 s	*Z* = 2.485 *p* = 0.013^*^
					Two-tail *p* = 0.344			
Shark 3	3	8.53 ± 0.85 s	8.90 ± 2.50 s	7: 3	One-tail *p* = 0.172	0 392–0.897	7.30 ± 2.71 s	*Z* = 1.421 *p* = 0.155
					Two-tail *p* = 0.344			
Shark 5	3	8.13 ± 0.61 s	7.50 ± 1.10 s	10: 0	One-tail *p* = 0.001^***^	0.679–1.000	5.20 ± 0.79 s	*Z* = 2.561 *p* = 0.010^**^
					Two-tail *p* = 0.002^**^			
Shark 6	4	6.85 ± 0.33 s	7.40 ± 0.95 s	6: 4	One-tail *p* = 0.377	0.312–0.833	7.50 ± 3.57 s	*Z* = 0.928 *p* = 0.353
					Two-tail *p* = 0.754			
Shark 7	5	8.66 ± 1.42 s	8.86 ± 2.02 s	8: 2	One-tail *p* = 0.055	0.479–0.954	7.60 ± 2.63 s	*Z* = 1.174 *p* = 0.240
					Two-tail *p* = 0.109			
Shark 8	9	7.61 ± 2.58 s	7.46 ± 1.92 s	4: 6	One-tail *p* = 0.377	0.167–0.688	6.40 ± 2.42 s	*Z* = 0.553 *p* = 0.580
					Two-tail *p* = 0.754			
Group	5.33 ± 2.52	7.46 ± 1.99 s	7.84 ± 1.75 s	42: 18	One-tail *p* = 0.001^***^	0.574–0.802	6.55 ± 2.50 s	*Z* = 3.703 *p* ≤ 0.001^***^
					Two-tail *p* = 0.002^**^			
**6 vs. 5 cm**								
Shark 3	3	8.23 ± 2.35 s	8.46 ± 2.45 s	7: 3	One-tail *p* = 0.172	0.392–0.897	11.80 ± 11.60 s	*Z* = 0.442 *p* = 0.658
					Two-tail *p* = 0.344			
Shark 5	3	7.00 ± 0.84 s	7.74 ± 1.85 s	10: 0	One-tail *p* = 0.001^***^	0.679–1.000	5.10 ± 2.51 s	*Z* = 2.517 *p* = 0.012^*^
					Two-tail *p* = 0.002^**^			
Shark 7	5	6.36 ± 0.82 s	8.30 ± 1.54 s	9: 1	One-tail *p* = 0.012^**^	0 574–>0.999	6.30 ± 2.41 s	*Z* = 1.924 *p* = 0.054
					Two-tail *p* = 0.002^**^			
Group	3.67 ± 1.16	6.90 ± 1.35 s	8.20 ± 1.95 s	26: 4	One-tail *p* ≤ 0.001^***^	0.697–0.953	7.73 ± 7.37 s	*Z* = 3.053 *p* = 0.002^**^
					Two-tail *p* = 0.001^***^			

p > 0.05 not significant,

(*) p ≤ 0.05 significant

(**) p ≤ 0.01 significant

(***) *p ≤ 0.001 significant*.

# Results are only shown for those individuals, who reached the learning criterion within 30 training sessions. Accordingly, group results refer only to these individuals.

**Table:** **Part 2: Statistics on regular training trials and transfer tests during Experiment 3: size pairs and Müller-Lyer deception (Experiments 3a and 3b)**.

**Subject^#^**	**Experiment 3b**								
	**Sessions to reach learning criterion**	**Average trial time [s] per regular training trial during training (Tr) and transfer (Tr6)**		**Sign and binomial test and 95% confidence interval [CI 95%] on absolute choices during transfers (T6)**				**Average trial time [s] per transfer trial (T6)**	**Mann-Whitney-U test on time differences [training (Tr6) ↔ transfers (T6)]**
		**Tr**	**Tr6**	**Left: right**	**Test**	**<-> vs. >-<**	**Test**		
Shark 3	17	8.08 ± 1.43 s	8.89 ± 2.25 s	14: 16	One-tail: *p* = 0.428	15: 15	One-tail: *p* = 0.572	6.93 ± 1.78 s	*Z* = 2.905 *p* = 0.004^**^
					Two-tail: *p* = 0.855		Two-tail: *p* = 1.145		
					CI 95% 0.302–0.639		CI 95% 0.302–0.637		
Shark 4	3	9.73 ± 0.85 s	9.08 ± 1.75 s	4: 26	One-tail: *p* ≤ 0.001^***^	13: 17	One-tail: *p* = 0.292	9.00 ± 2.16 s	*Z* = 0.035 *p* = 0.972
					Two-tail: *p* ≤ 0.001^***^		Two-tail: *p* = 0.585		
					CI 95% 0.047–0.303		CI 95% 0.274–0.608		
Shark 5	15	5.42 ± 0.88 s	6.41 ± 0.78 s	29: 1	One-tail: *p* ≤ 0.001^***^	16: 14	One-tail: *p* = 0.428	4.73 ± 1.28 s	*Z* = 4.434 *p* ≤ 0.001^***^
					Two-tail: *p* ≤ 0.001^***^		Two-tail: *p* = 0.855		
					CI 95% 0.819–>0.999		CI 95% 0.361–0.698		
Shark 6	6	10.88 ± 4.97 s	8.31 ± 1.45 s	25: 5	One-tail: *p* ≤ 0.001^***^	10: 20	One-tail: *p* = 0.049^*^	7.70 ± 3.73 s	*Z* = 1.463 *p* = 0.144
					Two-tail: *p* ≤ 0.001^***^		Two-tail: *p* = 0.099		
					CI 95% 0.959–0.931		CI 95% 0.191–0.513		
Shark 7	3	6.77 ± 0.49 s	10.15 ± 4.51 s	13: 17	One-tail: *p* = 0.295	18: 12	One-tail: *p* = 0.181	6.93 ± 1.74 s	*Z* = 2.929 *p* ≤ 0.001^***^
					Two-tail: *p* = 0.585		Two-tail: *p* = 0.362		
					CI 95% 0.274–0.608		CI 95% 0.423–0.754		
Shark 8	4	5.45 ± 0.40 s	6.94 ± 2.34 s	16: 14	One-tail: *p* = 0.428	15: 15	One-tail: *p* = 0.572	6.03 ± 2.16 s	*Z* = 1.027 *p* = 0.303
					Two-tail: *p* = 0.855		Two-tail: *p* = 1.144		
					CI 95% 0.361–0.698		CI 95% 0.331–0.668		
Group	8.00 ± 6.32	7.72 ± 2.26 s	8.29 ± 1.39 s	101: 79	One-tail: *p* = 0.057	87: 93	One-tail: *p* = 0.355	6.89 ± 1.45 s	*Z* = 4.412 *p* ≤ 0.001^***^
					Two-tail: *p* = 0.117		Two-tail: *p* = 0.709		
					CI 95% 0.488–0.632		CI 95% 0.414–0.556		

p > 0.05 not significant,

(*) p ≤ 0.05 significant

(**) p ≤ 0.01 significant

(***) p ≤ 0.001 significant

CI 95% = 95% confidence interval of a proportion.

# Results are only shown for those individuals, who reached the learning criterion within 30 training sessions. Accordingly, group results refer only to these individuals.

**Table 4:** **Sign and binomial test and 95% confidence interval to determine if those sharks, who did not reach the learning criterion within 30 sessions for single size pairs (Experiment 3a) or the Müller-Lyer deception (Experiment 3b) chose the positive (rewarded) stimulus significantly more often than the negative (unrewarded) stimulus**.

**Subject**	**Task**	**No. of sessions**	**No. of successful trials**	**One-tail *p*-value**	**Two-tail *p*-value**	**The 95% confidence interval extends from**
Shark 4	Size ratio 6–3 cm	30	126	0.003^**^	0.007^*^	0.3655–0.476
Shark 9	Size ratio 6–3 cm	30	134	0.037^*^	0.073	0.391–0.503
Shark 2	Size ratio 6–5 cm	30	138	0.092	0.184	0.404–0.517
Shark 6	Size ratio 6–5 cm	30	122	≤0.001^***^	0.002^**^	0 353–0.463
Shark 8	Size ratio 6–5 cm	30	135	0.047^*^	0.094	0.375–0.507
Shark 3	Size ratio 6–5.5 cm	30	141	0.163	0.326	0.414–0.526
Shark 5	Size ratio 6–5.5 cm	30	142	0.386	0.386	0.417–0.529
Shark 7	Size ratio 6–5.5 cm	30	137	0.074	0.149	0.421–0.533
Shark 2	Müller-Lyer deception	30	143	0.226	0.453	0.421–0.533
Shark 9	Müller-Lyer deception	30	135	0.047^*^	0.094	0.375–0.507

p > 0.05 not significant,

(*) p ≤ 0.05 significant

(**) p ≤ 0.01 significant

(***) *p ≤ 0.001 significant*.

#### Experiment 3a

Six out of eight sharks completed training of the first size pair (6 vs. 3 cm) successfully. On average it took 6.17 ± 3.37 sessions to reach the learning criterion, and a decision was made within 7.20 ± 1.44 s per trial (Table 3). Two sharks (Shark 4 and Shark 9) were not able to solve the task (Table 4) and were therefore excluded from further training and testing. During T5 transfers (5 vs. 5 cm), only Shark 6 as well as all sharks grouped together showed a significant side preference (Table 3). There were no significant differences between regular and transfer trial times for five out of six sharks (Shark 6) but for the whole group combined (Table 3).

All sharks, which were successful in solving the first size pair, were also able to complete training on the second (6 vs. 4 cm). On average 5.33 ± 2.52 sessions were needed to reach the learning criterion (Table 3). On average a decision was made within 7.46 ± 1.92 s per trial (Table 3).

During transfers (5 vs. 5 cm), only Shark 5 as well as all sharks grouped together (Table 3) showed a significant side preference. There was also a significant difference for two sharks (Shark 2, Shark 5) as well as for the whole group regarding average trial time i.e., regular vs. transfer test trials (Table 3).

Three out of six sharks, which were successful in solving the second size pair were also able to complete training on the third one (6 vs. 5 cm). On average 3.67 ± 1.16 sessions were needed to reach the learning criterion (Table 3). A decision was made on average within 6.90 ± 1.35 s per training trial (Table 3). Three sharks (Shark 2, Shark 6, Shark 8) were not able to solve the task (Table 4), and were excluded from further training. Shark 6 preferred the negative stimulus (i.e., the shorter of the two lines) significantly over the positive one.

During T5 transfer tests (5 vs. 5 cm), all but one shark (Shark 3) and all sharks grouped together showed a significant side preference (Shark 5, Shark 7, Table 3). There was no significant difference for any but one shark (Shark 5) as well as for the whole group in the average trial time to solve the regular training trials or the transfer test trials (Table 3).

None of the three sharks, which were successful in solving the third size pair, was able to complete training on the fourth size pair (6 vs. 5.5 cm) within the allocated 30 training sessions (Table 4).

#### Experiment 3b

Six out of eight sharks completed training on two lines of varying lengths featuring differently oriented arrowheads (Figure 4) successfully. On average 8.00 ± 6.32 sessions were needed to reach the learning criterion (Table 3; group results only refer to those individuals, who reached the learning criterion within 30 training sessions). A decision was made on average within 7.72 ± 2.26 s per training trial (Table 3). Two sharks (Shark 2, Shark 9) did not solve the task (Table 4), and were excluded from further training. During T6 transfers (ML deception: two center lines of equal length with differently oriented arrowheads, Figure 4), three out of six sharks (Shark 4, Shark 5, Shark 6, Table 3) showed a significant side preference. In contrast, only one shark showed a distinct preference for the inverted arrowheads (Shark 6, Table 3). Three sharks (Shark 3, Shark 5, Shark 7) as well as all sharks grouped together showed significantly different average trial times to solve the transfer compared to the regular trials (Table 3).

## Discussion

The visual experience of a line or an edge usually corresponds to a discontinuity in the intensity, wavelength, or spectral composition of the radiation that stimulates two contiguous areas of the retina (Kanizsa, 1974). The visual system accomplishes the organization of these contextual interactions by processing sensory information about shape, color, distance, and movement of objects according to its own rules (Kandel et al., 2000). Thus, form perception and the underlying neuronal mechanisms require a general representation of object boundaries, independent of how they are defined (Nieder and Wagner, 1999). Contour detecting cells within the visual system are unlikely to account for this phenomenon, but rather the subjective surface is generated by a visual system that has a tendency to complete certain figural elements (Kanizsa, 1976; Gerbino and Salmaso, 1987; Purghé and Coren, 1992). The brain appears to have expectations derived from both experience and intrinsic wiring for vision that form the basis for the assumptions it makes about what is to be seen in the visual world (Kanizsa, 1979; Day and Kasperczyk, 1983; Kandel et al., 2000).

One of the visual abilities essential to form perception is the reconstruction of contours absent from the retinal image (Nieder and Wagner, 1999) and the brain's association of certain parts of a scene to form a recognizable object while downgrading other parts (Kandel et al., 2000). Optical illusions demonstrate certain organizational mechanisms of visual perception and are known to be closely related to cortical processes in different vertebrates, such as humans (Bertenthal et al., 1980; Wede, 2008), cats (Bravo et al., 1988; De Weerd et al., 1990), monkeys (Vallortigara, 2004, 2008; Nielsen et al., 2006, 2008), owls (Nieder and Wagner, 1999; Nieder, 2002), and chickens (Vallortigara, 2006). There are several indications that parts of the fish telencephalon, such as the lateral and medial pallium could be considered as homologous to parts of the mammalian telencephalon, such as the hippocampus and the amygdala, (e.g., Northcutt, 1977, 1981, 1995; Salas et al., 1996a, 2003; Wullimann and Mueller, 2004; Durán et al., 2008, 2010; Nieuwenhuys, 2009; Martín et al., 2011). Nonetheless, other brain regions, such as the midbrain (e.g., in pigeons) may be involved in processing of illusionary contours as well (Niu et al., 2006).

Several aspects regarding the perception of optical illusions, such as the ability to reconstruct incomplete, partly occluded objects or subjective contours have already been successfully tested in a range of teleosts (e.g., Schuster and Amtsfeld, 2002; Wyzisk and Neumeyer, 2007; Sovrano and Bisazza, 2008, 2009; Siebeck et al., 2009). The present study aimed to behaviorally investigate the perception of Kanizsa figures (experiment 1), subjective contours (experiment 2), and the perception of the ML deceptions (experiment 3) in juvenile gray bamboo sharks (*Chiloscyllium griseum*).

Sharks needed on average ten sessions in the first and eight sessions in the second part of experiment 1 to discriminate successfully between squares and triangles. During the following two sets of transfer tests, all but one shark chose the corresponding Kanizsa figure significantly more often than any of the seven different randomized Pacmen figures (Table 1) that were presented as alternatives. All but one shark significantly preferred the Kanizsa figure, which most closely resembled the positive training stimulus. While other factors, such as symmetry features of the Pacmen figures could have potentially influenced the choosing process in the transfer tests of experiment 1a, the results of the transfer trials in experiment 1b clearly show that this was not the deciding criterion implemented by sharks. This data, indicating that Kanizsa figures were easily perceived as squares and triangles, was supported by the data collected on trial time; there was no significant difference in the average trial time needed to make a definite choice during the regular training trials (choosing between two “real” symbols) compared to the transfer trials (choosing between Kanizsa figures; Figure 2, Table 1). Although group 2 performed slightly better than group 1, the recognition and differentiation of square-shaped as well as triangle-shaped Kanizsa figures was equally effective for both groups (Table 1). Results clearly show that sharks can perceive Kanizsa figures. As in humans, images of the Kanizsa squares or triangles had to emerge from fictional contours supplied by the brain, pointing to a similar or analogical organizational mechanism of visual perception to the “filling-in” mechanism found in mammals (Kellman et al., 1998; Kandel et al., 2000). Comparable results were also found in goldfish, *Carassius auratus* (Wyzisk, 2005). However, in the goldfish, square and triangle discriminations seemed to be based on very specific features of these forms, since not the entire figure was needed to retain the discrimination ability.

In experiment 2 sharks chose a white square presented on white diagonal lines over a rhomboid within 11.13 ± 8.44 sessions (Table 2). During T3 transfer tests, sharks had to choose the subjective contour defining a square by using grating gaps within the white lines. All but one shark chose the correct subjective contour representing a square significantly more often than the trained negative stimulus representing a rhomboid (Table 2). When facing subjective contours defining a square by using phase-shifted abutting gratings (T4 tests), all sharks maintained the high level of performance of the first transfer tests (Table 2). Again, all but one shark appeared to implement easily what they had learned during training. This is supported by the nearly constant average trial times during T3 and T4 transfer trials compared to regular training trials. The results indicate that sharks are capable of perceiving subjective contours as shown previously also for redtail splitfins (*Xenotoca eiseni*, Sovrano and Bisazza, 2009), barn owls (*Tyto alba*, Nieder and Wagner, 1999; Nieder, 2002), chickens (Vallortigara, 2006), and primates (Vallortigara, 2004, 2008). Barn owls, for example, which were trained to discriminate between two real shapes, were also able to distinguish between the corresponding illusionary contours and showed a clear preference for the positive training stimulus. Nieder and Wagner concluded that the birds recognized the illusionary contours as “true” objects by “filling-in” the missing edges. Surprisingly, goldfish were unable to recognize phase-shifted illusionary squares (Wyzisk and Neumeyer, 2007); however, results of this study could have been negatively influenced by methodological errors regarding the line sizing (Sovrano and Bisazza, 2009).

Considering the combined results of the first two experiments, it seems unlikely that the sharks focused on single feature elements of the stimulus, such as edges or lines instead of the overall shape. Interruptions and boundary discontinuities for example were present in both stimuli (i.e., Kanizsa figures and subjective contours with grating gaps or phase-shifted abutting gratings) and could have not aided in the discrimination process. Instead, it is likely that sharks applied the concepts of “filling-in” (Kandel et al., 2000) or “(a)modal completion” (Michotte et al., 1964/1991; Kanizsa et al., 1993; Singh, 2004) which occurs when parts of an object are camouflaged by an overlying surface, which projects the same luminance and color as the nearer object (Singh, 2004). In case of the Kanizsa figures, the “incomplete” Pacmen figures appeared as fully-uninterrupted circles, partially hidden behind an occluding figure. In case of the subjective contours with grating gaps or phase-shifted abutting gratings, a continuous square (or rhomboid) was recognized on a background of white diagonal lines (i.e., completing the lines amodally behind the illusory surface, Michotte et al., 1964/1991; Kanizsa et al., 1993).

Goldfish (Wyzisk and Neumeyer, 2007) and redtail splitfins (Sovrano and Bisazza, 2008) can recognize and “mentally complete” partly occluded objects, which represents another form of amodal completion. Sovrano and Bisazza (2008) trained redtail splitfins to discriminate between a complete and an amputated disc. The fish then performed in test trials in which hexagonal polygons produced or averted the impression of a partial occlusion of the disk. Fish behaved as if they were experiencing visual completion of the partly occluded stimuli (Sovrano and Bisazza, 2008). The perception of amodal completion and the perception of subjective contours both seem to use the same basic mechanisms to deal with occlusion problems (Kellman and Shipley, 1991; Kellman et al., 2001, 2005).

In preparation for the ML deception, training involved the discrimination of two lines of different length (experiment 3). As the ML deception evokes only a slight, not very pronounced size illusion, the difference in length between the two lines was reduced gradually with continuous training. Six out of eight sharks were able to significantly often select the longer of the two lines in two size pairs (6 vs. 3 cm, 6 vs. 4 cm) within 6 and 5 sessions, respectively (Table 3). Three sharks even discriminated 6 vs. 5 cm within 4 sessions (Table 3). In this task, sharks performed much better than goldfish (Wyzisk, 2005); these decided at chance level (50% correct) when being presented with lines of 5 vs. 3 cm, 6 vs. 4 cm, or 5 vs. 2 cm. When being presented with two lines of equal length (T5), sharks chose according to chance level or developed side preferences (Table 3). In the following task, sharks were presented with two lines of varying lengths (6 vs. 3 cm) randomly featuring differently oriented ends (either two arrowheads or -tails, Figure 4). Six sharks were able to reach the learning criterion on average within 8 sessions.

In the ML deception, two center lines of equal length, one featuring two inverted and the other two normal arrowheads, appear to be unequal in length due to the differently oriented arrowheads, which evoke a spatial impression. Humans judge the size of an object by comparing it to its immediate surrounding; thus, the spatial relationship of objects helps to interpret the image. Humans perceive the lines to be unequal because the brain uses shape and the experience from the spatial sense as an indicator of sizing (Kandel et al., 2000). As typical for many illusions, knowing that the lines are equal does not prevent humans from being misled by this illusion (Kandel et al., 2000). Surprisingly though, not all human cultures react equally to these illusions (Rivers, 1901), with Europeans being more susceptive than cultures such as Inuits, Aborigines or Africans (Segall et al., 1966; Berry, 1968). Most likely, several factors, such as eye pigmentation or enhancement though a “carpentered” environment contribute to these intercultural differences (Jahoda, 1971). The obtained results of the present study revealed a very different response to the ML deception than expected or found in most humans. Surprisingly, the sharks were not tricked by the “length-confusion” but displayed the same behavior as found when lines of equal length (featuring no or randomly oriented arrowheads- and tails; T6 tests) were presented. While three sharks developed a significant side preference (the other three chose according to chance level), only one shark showed a distinct tendency for a specific arrow (i.e., arrowtails, Table 3). For some unknown reason, three sharks as well as the whole group made their choice significantly faster during the transfer tests compared to regular trials (Table 3). Overall though, sharks seemed to identify the length of the center lines, irrespective of the surrounding elements. Thus, the here presented results on sharks are consistent with the results found in goldfish during an earlier study (Wyzisk, 2005) and stand in contrast to results obtained from other species such as gray parrots (Pepperberg et al., 2008), pigeons (Nakamura et al., 2006), chickens (Winslow, 1933), ring doves (Warden and Baar, 1929), capuchin monkeys (Suganuma et al., 2007), and rhesus macaques (Tudusciuc and Nieder, 2010).

Potentially, results could have been different in case other versions of the Müller-Lyer illusion had been tested, such as the Brentano variation (as used e.g., by Pepperberg et al., 2008; variation in the lengths or thickness of the center lines or the angle of the arrows or both). The present study obviously cannot exclude this, but the original version of the ML illusion that was tested here was not perceived. As potential mechanisms were not investigated any further it is impossible to decide which strategies may have been used. However, seeing oriented line terminations in the stimuli is not the same as perceiving an illusory contour. In fact, in all experiments sharks had to pay attention to the length of the lines, not to the orientation of arrows. Illusionary trials (i.e., T6) were randomly interspersed with regular training trials, featuring lines of different length with arrowheads and tails and results were always significant in those trials. Accordingly, sharks did not look for anything but the line length and all sharks with the exception of one were proven not to pay attention to the arrow-formation. All other sharks showed side preferences, a common response if animals do not know what to choose. As there was no difference in the length of the lines, it is irrelevant if sharks have low or high visual acuity.

During experiment 3a, lines of 6 vs. 5 cm were still told apart from each other by some sharks—which would have about equaled the length difference between the two versions shown in the Müller Lyer tests in experiment 3b (including the arrowheads, not just the center lines). So if acuity was good enough to distinguish 6 and 5 cm (experiment 3a), then it should have been good enough to distinguish the length of the illusionary figures (experiment 3b, T6)—at least in some animals. The homogenous response that none of the sharks solved the task clearly indicates that no difference as observed as there was none. This recalls the fact that not all humans perceive the ML illusion (as it evokes only a very slight deception) and not all humans react equally to it (Rivers, 1901; Segall et al., 1966; Berry, 1968).

Visual perception is a creative process—not only in humans, mammals, birds and teleosts but also in bamboo sharks, a representative of one of the oldest vertebrate groups. Present results not only reveal that bamboo sharks have the ability to perceive or reject optical illusions. Moreover, they provide information on the evolutionary origin and development of selected cognitive abilities and the characteristics of shared or non-shared neural mechanisms. Lastly, as found in other cognition experiments, present results highlight the behavioral variability found among individuals trained in the same procedure and using the same training schedule. The often observed, apparently erratic nature of the individual learning success is part of this variability, as well as the sharks' different capabilities regarding the perception of optical illusions.

### Conflict of interest statement

The authors declare that the research was conducted in the absence of any commercial or financial relationships that could be construed as a potential conflict of interest.

## References

[B1] Agrillo C. Petrazzini M. E. M. Dadda M. (2013). Illusionary patterns are fishy for fish, too. Front. Neural Circuits 7:137 10.3389/fncir.2013.00137 24009560 PMC3755263

[B2] Berry J. W. (1968). Ecology, perceptual development and the Muller-Lyer illusion. Br. J. Psychol. 59, 205–210 10.1111/j.2044-8295.1968.tb01134.x 5760069

[B3] Bertenthal B. I. Campos J. J. Haith M. M. (1980). Development of visual organization: the perception of subjective contours. Child Dev. 51, 1072–1080 10.2307/1129546 7471916

[B4] Bravo M. Blake R. Morrison S. (1988). Cats see subjective contours. Vision Res. 28, 861–865 10.1016/0042-6989(88)90095-8 3250081

[B5] Brown C. Laland K. Krause J. (2011). Fish cognition and behaviour, in Fish Cognition and Behaviour, 2nd Edn, eds Brown C. Laland K. Krause J. (Cambridge, UK: Wiley Publishing), 1–8

[B6] Compagno L. J. V. Dando M. Fowler S. (2005). A Field Guide to the Sharks of the World. London: Collins

[B7] Darmaillacq A. S. Dickel L. Rahmani N. Shashar N. (2011). Do reef fish, *Variola louti* and *Scarus niger*, perform amodal completion? Evidence from a field study. J. Comp. Psychol. 125, 273 10.1037/a0024295 21842982

[B8] Day R. H. Kasperczyk R. T. (1983). Amodal completion as a basis for illusory contours. Percept. Psychophys. 33, 355–364 10.3758/BF03205882 6866697

[B9] De Weerd P. Vandenbussche E. De Bruyn B. Orban G. A. (1990). Illusory contour orientation discrimination in the cat. Behav. Brain Res. 39, 1–17 10.1016/0166-4328(90)90117-W 2390193

[B10] Durán E. Ocaña F. M. Broglio C. Rodríguez F. Salas C. (2010). Lateral but not medial telencephalic pallium ablation impairs the use of goldfish spatial allocentric strategies in a “hole-board” task. Behav. Brain Res. 214, 480–487 10.1016/j.bbr.2010.06.010 20600353

[B11] Durán E. Ocaña F. M. Gómez A. Jiménez-Moya F. Broglio C. Rodríguez F. (2008). Telencephalon ablation impairs goldfish allocentric spatial learning in a hole-board task. Acta Neurobiol. Exp. 68, 519–525 19112476 10.55782/ane-2008-1719

[B12] Fuss T. Bleckmann H. Schluessel V. (2014a). The shark *Chiloscyllium griseum* can orient using turn responses before and after partial telencephalon ablation. J. Comp. Physiol. A Neuroethol. Sens. Neural Behav. Physiol. 200, 19–35 10.1007/s00359-013-0858-y 24114617

[B13] Fuss T. Bleckmann H. Schluessel V. (2014b). Place learning prior to and after telencephalon ablation in bamboo and coral cat sharks (*Chiloscyllium griseum* and *Atelomycterus marmoratus*). J. Comp. Physiol. A Neuroethol. Sens. Neural Behav. Physiol. 200, 37–52 10.1007/s00359-013-0859-x 24114618

[B14] Fuss T. Bleckmann H. Schluessel V. (2014c). Visual discrimination abilities in the gray bamboo shark (*Chiloscyllium griseum*). Zoology. [Epub ahead of print]. 10.1016/j.zool.2013.10.009 24369760

[B15] Gerbino W. Salmaso D. (1987). The effect of amodal completion on visual matching. Acta Psychol. 65, 25–46 10.1016/0001-6918(87)90045-X 3618293

[B70] Gregory R. L. (1972). Cognitive contours. Nature 238, 51–52 10.1038/238051a0 12635278

[B17] Guttridge T. L. Myrberg A. A. Porcher I. F. Sims D. W. Krause J. (2010). The role of learning in shark behavior. Fish Fish. 10, 450–469 10.1111/j.1467-2979.2009.00339.x

[B71] Hart N. S. Theiss S. M. Harahush B. K. Collin S. P. (2011). Microspectrophotometric evidence for cone monochromacy in sharks. Naturwissenschaften 98, 193–201 10.1007/s00114-010-0758-8 21212930

[B16] Jahoda G. (1971). Retinal pigmentation, illusion susceptibility and space perception. Int. J. Psychol. 6, 199–207 10.1080/00207597108246683656735

[B18] Kandel E. R. Schwartz J. H. Jessell T. M. (2000). Principles of Neural Science. New York, NY: McGraw-Hill

[B19] Kanizsa G. (1974). Contours without gradients or cognitive contours? G. Ital. Psicol. 1, 93–113

[B20] Kanizsa G. (1976). Subjective contours. Sci. Am. 234, 48–52 10.1038/scientificamerican0476-48 1257734

[B72] Kanizsa G. (1979). Organization in Vision: Essays on Gestalt Perception. New York, NY: Praeger

[B21] Kanizsa G. Renzi P. Conte S. Compostela C. Guerani L. (1993). Amodal completion in mouse vision. Perception 22, 713–721 10.1068/p220713 8255701

[B22] Kellman P. J. Garrigan P. Shipley T. F. (2005). Object interpolation in three dimesions. Psychol. Rev. 112, 586–609 10.1037/0033-295X.112.3.586 16060752

[B23] Kellman P. J. Guttman S. Wickens T. (2001). Geometric and neural models of contour and surface interpolation in visual object perception, in From Fragments to Objects: Segmentation and Grouping in Vision, eds Shipley T. F. Kellman P. J. (New York, NY: Elsevier Science), 183–245 10.1016/S0166-4115(01)80027-3

[B24] Kellman P. J. Shipley T. F. (1991). A theory of visual interpolation in object perception. Cogn. Psychol. 23, 141–221 10.1016/0010-0285(91)90009-D 2055000

[B25] Kellman P. J. Yin C. Shipley T. F. (1998). A common mechanism for illusory and occluded object completion. J. Exp. Psychol. Hum. Percept. Perform. 24, 859–869 10.1037/0096-1523.24.3.859 9627421

[B26] Kimber J. A. Sims D. W. Bellamy P. B. Gill A. B. (2014). Elasmobranch cognitive ability: using electroreceptive foraging behaviour to demonstrate learning, habituation and memory in a benthic shark. Anim. Cogn. 17, 55–65 10.1007/s10071-013-0637-8 23620366

[B27] Kuba M. J. Byrne R. A. Burghardt G. M. (2010). A new method for studying problem solving and tool use in stingrays (Potamotrygon castexi). Anim. Cogn. 13, 507–513 10.1007/s10071-009-0301-5 20020169

[B28] Martín I. Gómez A. Salas C. Puerto A. Rodríguez F. (2011). Dorsomedial pallium lesions impair taste aversion learning in goldfish. Neurobiol. Learn. Mem. 96, 297–305 10.1016/j.nlm.2011.06.003 21689770

[B29] Michotte A. Thinés G. Crabbé G. (1964/1991). Amodal completion of perceptual structures, in Michotte's Experimental Phenomenology of Perception (Original work published 1964), eds Thinés G. Costall A. Butterworth G. (New York, NY: Erlbaum, Hillsdale), 140–167

[B30] Nakamura N. Fujita K. Ushitani T. Miyatat H. (2006). Perception of the standard and the reversed Müller-Lyer figures in pigeons (*Columba livia*) and humans (*Homo sapiens*). J. Comp. Psychol. 120, 252–261 10.1037/0735-7036.120.3.252 16893262

[B31] Nieder A. (2002). Seeing more than meets the eye: processing of illusory contours in animals. J. Comp. Physiol. A Neuroethol. Sens. Neural Behav. Physiol. 188, 249–260 10.1007/s00359-002-0306-x 12012096

[B32] Nieder A. Wagner H. (1999). Perception and neuronal coding of subjective contours in the owl. Nat. Neurosci. 2, 660–663 10.1038/10217 10404200

[B33] Nielsen K. J. Logothetis N. K. Rainer G. (2006). Discrimination strategies of humans and rhesus monkeys for complex visual displays. Curr. Biol. 16, 814–820 10.1016/j.cub.2006.03.027 16631590

[B34] Nielsen K. J. Logothetis N. K. Rainer G. (2008). Object features used by humans and monkeys to identify rotated shapes. J. Vis. 8, 1–15 10.1167/8.2.9 18318635

[B35] Nieuwenhuys R. (2009). The forebrain of actinopterygians revisited. Brain Behav. Evol. 73, 229–252 10.1159/000225622 19546532

[B73] Niu Y.-Q. Xiao Q. Liu R.-F. Wu L.-Q. Wang S.-R. (2006). Response characteristics of the pigeon's pretectal neurons to illusory contours and motion. J. Physiol. 577, 805–813 10.1113/jphysiol.2006.120071 17038429 PMC1890384

[B36] Northcutt R. G. (1977). Elasmobranch central nervous system organization and its possible evolutionary significance. Am. Zool. 17, 411–429

[B37] Northcutt R. G. (1981). Evolution of the telencephalon in nonmammals. Annu. Rev. Neurosci. 4, 301–350 10.1146/annurev.ne.04.030181.001505 7013637

[B38] Northcutt R. G. (1995). The forebrain of gnathostomes: in search of a morphotype. Brain Behav. Evol. 46, 275–318 10.1159/000113279 8564468

[B39] Paradiso M. A. Shimojo S. Nakayama K. (1989). Subjective contours, tilt aftereffects, and visual cortical organization. Vision Res. 29, 1205–1213 10.1016/0042-6989(89)90066-7 2617866

[B40] Pepperberg I. Vicinay J. Cavanagh P. (2008). Processing of the Müller-Lyer illusion by a Grey parrot (*Psittacus erithacus*). Perception 37, 765–781 10.1068/p5898 18605149

[B41] Petry S. Meyer G. E. (1987). The Perception of Illusory Contours. New York, NY: Springer Verlag 10.1007/978-1-4612-4760-9

[B42] Purghé F. Coren S. (1992). Amodal completion, depth stratification, and illusory figures: a test of Kanizsa's explanation. Perception 21, 325–335 10.1068/p210325 1437451

[B43] Rivers W. H. R. (1901). Introduction and vision, in Reports of the Cambridge Anthropological Expedition to the Torres Straits, Vol. 11, Part I. ed Haddon A. C. (Cambridge: University Press), 152–153

[B44] Rock I. Anson R. (1979). Illusory contours as the solution to a problem. Perception 8, 665–681 10.1068/p080665 530808

[B45] Salas C. Broglio C. Rodríguez F. (2003). Evolution of forebrain and spatial cognition in vertebrates: conservation across diversity. Brain Behav. Evol. 62, 72–82 10.1159/000072438 12937346

[B46] Salas C. Rodríguez F. Vargas J. P. Durán E. Torres B. (1996a). Spatial learning and memory deficits after telencephalic ablation in goldfish trained in place and turn maze procedures. Behav. Neurosci. 110, 965–980 10.1037/0735-7044.110.5.965 8918999

[B47] Schluessel V. Bleckmann H. (2005). Spatial memory and orientation strategies in the elasmobranch Potamotrygon motoro. J. Comp. Physiol. A Neuroethol. Sens. Neural Behav. Physiol. 191, 695–706 10.1007/s00359-005-0625-9 15895237

[B48] Schluessel V. Bleckmann H. (2012). Memory retention in the grey bamboo shark *Chiloscyllium griseum*. Zoology 115, 346–353 10.1016/j.zool.2012.05.001 23040178

[B49] Schumann F. (1900). Beiträge zur analyse der gesichtswahrnehmungen. Z. Psychol. 23, 1–32

[B50] Schuster S. Amtsfeld S. (2002). Template-matching describes visual pattern-recognition tasks in the weakly electric fish *Gnathonemus petersii*. J. Exp. Biol. 205, 549–557 11893769 10.1242/jeb.205.4.549

[B51] Schwarze S. Bleckmann H. Schluessel V. (2013). Avoidance conditioning in bamboo sharks (*Chiloscyllium griseum* and *C. punctatum)*: behavioral and neuroanatomical aspects. J. Comp. Physiol. A Neuroethol. Sens. Neural Behav. Physiol. 199, 843–856 10.1007/s00359-013-0847-1 23958858

[B52] Segall M. H. Campbell L. T. Herskovits M. J. (1966). The Influence of Culture on Visual Perception. Indianapolis, IN: Bobbs-Merrill

[B53] Siebeck U. Litherland L. Wallis G. (2009). Shape learning and discrimination in reef fish. J. Exp. Biol. 212(Pt 13), 2113–2119 10.1242/jeb.028936 19525438

[B54] Singh M. (2004). Modal and amodal completion generate different shapes. Psychol. Sci. 15, 454–459 10.1111/j.0956-7976.2004.00701.x 15200629

[B55] Sovrano V. A. Bisazza A. (2008). Recognition of partly occluded objects by fish. Anim. Cogn. 11, 161–166 10.1007/s10071-007-0100-9 17636365

[B56] Sovrano V. A. Bisazza A. (2009). Perception of subjective contours in fish. Perception 38, 579–590 10.1068/p6121 19522325

[B57] Spaet J. L. Y. Kessel S. T. Gruber S. H. (2010). Learned hook avoidance of lemon sharks (*Negaprion brevirostris*) based on electroreception and shock treatment. Mar. Biol. Res. 6, 399–407 10.1080/17451000903039749

[B58] Suganuma E. Pessoa V. F. Monge-Fuentes V. Castro B. M. Tavares M. C. H. (2007). Perception of the Müller-Lyer illusion in capuchin monkeys (*Cebus apella*). Behav. Brain Res. 182, 67–72 10.1016/j.bbr.2007.05.014 17586063

[B59] Tudusciuc O. Nieder A. (2010). Comparison of length judgements and the Müller-Lyer illusion in monkeys and humans. Exp. Brain Res. 207, 221–231 10.1007/s00221-010-2452-7 20972775

[B60] Vallortigara G. (2004). Visual cognition and representation in birds and primates, in Vertebrate Comparative Cognition: Experimental Explorations of Animal Intelligence, eds Rogers L. J. Kaplan G. (New York, NY: Kluwer Academic/Plenum), 57–94 10.1007/978-1-4419-8913-0_2

[B61] Vallortigara G. (2006). The cognitive chicken: visual and spatial cognition in a non-mammalian brain, in Comparative Cognition: Experimental Explorations of Animal Intelligence eds Wasserman E. A. Zentall T. R. (Oxford: Oxford University Press), 41–58

[B62] Vallortigara G. (2008). Animals as natural geometers, in Cognitive Biology: Evolutionary and developmental Perspectives on Mind, Brain and Behavior, eds Tommasi L. Nadel L. Peterson M. (Cambridge, MA: MIT Press), 83–104

[B63] von der Heydt R. (1995). Form analysis in visual cortex. Cogn. Neurosci. 365–382 16046147

[B64] Warden D. J. Baar J. (1929). The Mü ller-Lyer illusion in the ring dove, *Turtur risorius*. J. Comp. Psychol. 9, 275–292 10.1037/h0071052

[B65] Wede J. (2008). The Effect of Attention on the Perception of Illusory Contours. Ph.D. thesis, Purdue University, West Lafayette, IN, USA

[B66] Winslow C. N. (1933). Visual illusions in the chick. Arch. Psychol. 153, 1–83 16970206

[B67] Wullimann M. F. Mueller T. (2004). Teleostean and mammalian forebrains contrasted: evidence from genes to behavior. J. Comp. Neurol. 475, 143–162 10.1002/cne.20183 15211457

[B68] Wyzisk K. (2005). Experimente zur Form- und Größenwahrnehmung beim Goldfisch (Carassius auratus) unter Verwendung von Scheinkonturen und Größentäuschungen. Ph.D. thesis, Johannes-Gutenberg-Universität Mainz, Germany

[B69] Wyzisk K. Neumeyer C. (2007). Perception of illusory surfaces and contours in goldfish. Vis. Neurosci. 24, 291–298 10.1017/S095252380707023X 17822573

